# Bovine Serum Albumin‐Based Sponges as Biocompatible Adsorbents: Development, Characterization, and Perfluorooctane Sulfonate Removal Efficiency

**DOI:** 10.1002/smsc.202400497

**Published:** 2025-03-02

**Authors:** Maria Kaeek, Yair Rajmiel, Ofek Goldreich, Luai R. Khoury

**Affiliations:** ^1^ Department of Materials Science and Engineering Technion Israel Institute of Technology Haifa 32000 Israel

**Keywords:** biocompatible materials, bovine serum albumin‐based sponges, Langmuir and Freundlich isotherms, microstructure optimizations, perfluorooctane sulfonate adsorptions, polyfluoroalkyl substances, protein‐based materials

## Abstract

Polyfluoroalkyl substances, particularly perfluorooctane sulfonate (PFOS), are persistent environmental pollutants with severe health risks due to their bioaccumulation and resistance to degradation. Current PFOS removal technologies are limited by either efficiency, cost, or environmental concerns. Here, we introduce a biocompatible, protein‐based sponge approach using bovine serum albumin (BSA) as a scaffold for efficient PFOS removal. We developed highly porous, mechanically robust sponges by optimizing foaming parameters such as mixing speed, duration, and surfactant concentration. Advanced characterization techniques, including microcomputed tomography and cryo‐scanning electron microscopy, confirmed the sponges’ structural integrity. Leveraging natural BSA–PFOS interactions, the sponges demonstrate effective PFOS removal, achieving up to ≈80% efficiency at a pH of ≈7.4, similar to natural water systems. Adsorption behavior is described using Langmuir and Freundlich isotherms, showing high adsorption capacity and surface interaction. Mechanical testing confirmed durability, making the sponges suitable for real‐world applications. This eco‐friendly method surpasses conventional PFOS removal techniques, offering a cost‐effective solution with potential applications in drug delivery, tissue engineering, and catalysis. This work paves the way for developing multifunctional, porous protein‐based materials that address urgent environmental challenges while offering versatile applications in biotechnology.

## Graphical abstract

A lightweight, porous bovine serum albumin (BSA)‐based sponge elegantly rests on a flower petal, visually emphasizing its delicate structure and minimal weight. The accompanying schematic illustrates the formation of BSA–perfluorooctane sulfonate (PFOS) complexes through hydrophobic interactions, electrostatic forces, and hydrogen bonding, highlighting the sponge's advanced functionality in efficiently adsorbing PFOS for environmental remediation.

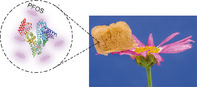

## 1 Introduction

Polyfluoroalkyl substances (PFASs), including compounds such as perfluorooctane sulfonate (PFOS; C_8_F_17_SO^−3^) and perfluorooctanoate (PFOA; C_8_F_15_COO^−^), are infamously known as “forever chemicals” due to their extreme persistence in the environment.^[^ ^1^, ^2^, ^3^ ^]^ Among these, PFOS has garnered particular attention due to its widespread use in consumer products, including textiles, polymers, food packaging, and industrial surfactants.^[^ ^4^, ^5^ ^]^ Its environmental persistence, lasting over 40 years in surface water,^[^ ^6^ ^]^ is primarily attributed to the strength of the C—F bond, which imparts significant resistance to degradation, leading to bioaccumulation across ecosystems.^[^ ^7^, ^8^, ^9^ ^]^ Exposure to PFOS through food, air, and water is increasingly recognized as a major health concern, with links to severe illnesses such as cancer, immune dysfunction, and hypertension.^[^ ^10^, ^11^, ^12^ ^]^ Unlike many other substances, PFOS does not accumulate in lipids but in protein‐rich tissues such as the liver, kidneys, and blood serum albumin,^[^ ^13^ ^]^ which is the primary binding target for PFASs, particularly PFOS (Ka ≈10^6^) under physiological conditions.^[^ ^14^, ^15^ ^]^

Intense efforts have been directed toward developing advanced water treatment technologies for PFOS removal, which is especially urgent given that PFOS concentrations in drinking water have been reported at 3.5 and up to 423 ppb in human blood.^[^ ^16^, ^17^ ^]^ These technologies aim to function efficiently across the typical pH range of natural and engineered systems, including freshwater ecosystems and biological fluids.^[^ ^18^, ^19^, ^20^ ^]^ Since common water sources like drinking water, sewage, and rivers generally have a pH range of 6.5 to 8.5,^[^ ^21^, ^22^, ^23^ ^]^ there is an urgent need for effective PFOS removal methods to prevent contamination and protect public health. Granular activated carbon (GAC) is frequently used in water treatment plants to reduce contaminants, including PFASs. However, GAC can sometimes result in pollutant transfer to another medium, posing a risk of further contamination.^[^ ^24^ ^]^ High‐pressure membrane processes such as nanofiltration and reverse osmosis have shown high removal efficiency for PFAS in water reclamation and treatment systems, but these methods involve significant energy and operational costs, potentially limiting their broader application.^[^ ^24^ ^]^ Meanwhile, advanced oxidation processes, including ozonation and photocatalytic degradation, have demonstrated high PFAS degradation efficiencies in both deionized and river water, though they often generate harmful byproducts and require precise control over reaction conditions, presenting challenges for real‐world applications.^[^ ^25^ ^]^

Since the 1990s, functional porous sponges have attracted substantial interest due to their high porosity, large surface area, low density, and lightweight characteristics.^[^ ^26^, ^27^, ^28^, ^29^ ^]^ These features, coupled with their functional versatility, have broadened their applications across fields such as catalysis,^[^ ^30^, ^31^, ^32^ ^]^ sensing,^[^ ^33^, ^34^, ^35^ ^]^ electronic devices,^[^ ^36^, ^37^, ^38^ ^]^ drug delivery systems,^[^ ^39^, ^40^, ^41^ ^]^ and tissue engineering scaffolds.^[^ ^42^, ^43^, ^44^, ^45^, ^46^ ^]^ Recently, protein‐based hydrogels have emerged as promising materials because of their inherent biocompatibility, structural diversity, and nanoscale mechanical properties.^[^ ^47^, ^48^, ^49^, ^50^, ^51^, ^52^, ^53^, ^54^, ^55^, ^56^, ^57^, ^58^, ^59^, ^60^, ^61^ ^]^

Despite these advancements, the development of protein‐based sponges remains challenging. For instance, oat protein‐derived sponges exhibited strength comparable to commercial polyurethane,^[^ ^62^ ^]^ while soy‐based sponges demonstrated diverse chemical functionality, amphoteric behavior, and pH responsiveness.^[^ ^63^ ^]^ Other examples include keratin‐based sponges, which mimic natural tissues and promote cell proliferation,^[^ ^64^ ^]^ as well as whey protein sponges, which have been employed to absorb both polar and nonpolar liquids.^[^ ^65^ ^]^ However, current synthesis methods often require extreme conditions, such as high temperatures (exceeding 70 °C) and acidic environments (pH ≈2), which can denature proteins and reduce functionality.^[^ ^66^, ^67^ ^]^ Additionally, these methods frequently fail to achieve adequate control over the sponges’ microstructure‐an essential factor in tailoring physical and chemical properties for specific applications.

To address these challenges, we developed a novel, biocompatible, and eco‐friendly sponge based on bovine serum albumin (BSA). Recognizing that PFOS accumulates in protein‐rich environments and binds strongly to BSA, we selected BSA for its natural compatibility and efficiency in PFOS adsorption.^[^ ^68^, ^69^, ^70^ ^]^ Our method utilizes a hydrogel mixture of BSA, ammonium persulfate (APS) as a polymerization initiator, ruthenium (II) tris‐bipyridyl dication (Ru(II)bpy_3_^+2^) as a low‐cytotoxicity crosslinker, and Tween‐20 (TW‐20), a commonly used biocompatible surfactant that enhances foam stability and pore structure. Vigorous mixing of this mixture produces a stable BSA‐based foam, which is then crosslinked under white light to covalently crosslink adjacent tyrosine residues,^[^ ^71^ ^]^ resulting in a BSA‐based sponge. The sponge's macro/microstructure and pore size distributions were characterized using microcomputed tomography (microCT), cryoscanning electron microscopy (cryo‐SEM), and porosimetry. To confirm the preservation of the protein's structural integrity and functionality, attenuated total reflection‐Fourier transform infrared (ATR‐FTIR) spectroscopy, X‐ray diffraction (XRD), and visual detection methods were employed, demonstrating successful retention of BSA's properties before and after sponge formation.

Our results show that the interplay between TW‐20 and BSA, along with their foam‐stabilizing effects, enables the production of protein‐based sponges with adjustable microstructures and diverse physical properties, such as density, water absorption capacity, and porosity. These sponges exhibited tailored mechanical properties, displaying stability and flexibility under compressive forces, which enhances their application potential. In addition, X‐ray photoelectron spectroscopy (XPS) measurements revealed the interactions between PFOS and various functional groups on BSA, affirming the sponge's capability to capture PFOS effectively. Therefore, our functional BSA‐based sponges demonstrated efficient PFOS adsorption across a range of concentrations, simulating conditions with a pH of ≈7.4—similar to those found in drinking and river water. In addition, adsorption behavior was well‐described by both Langmuir and Freundlich isotherm models. This work highlights the formation of a novel, functional, and biocompatible sponge material with controlled microstructure, emphasizing its potential for environmental remediation, drug delivery, tissue engineering, and catalysis.

## 2 Results and Discussion

### 2.1 Characterization of BSA‐Based Sponges: Influence of Foaming Dynamics on Microstructure, Porosity, and Mechanical Properties

To develop BSA‐based sponges, we used TW‐20 as the primary foaming agent. TW‐20 is a non‐ionic surfactant with excellent water solubility and superior foaming properties compared to other surfactants in the Tween family.^[^ ^72^, ^73^ ^]^ Its presence, alongside BSA in the foaming system, enhances interactions between the amphiphilic molecules, forming mixed mobile and immobile layers at the foam surface. This cooperative protein‐surfactant interaction plays a key role in tailoring the microstructure, functionality, and mechanical properties of the protein‐based sponges.

**Figure 1:**
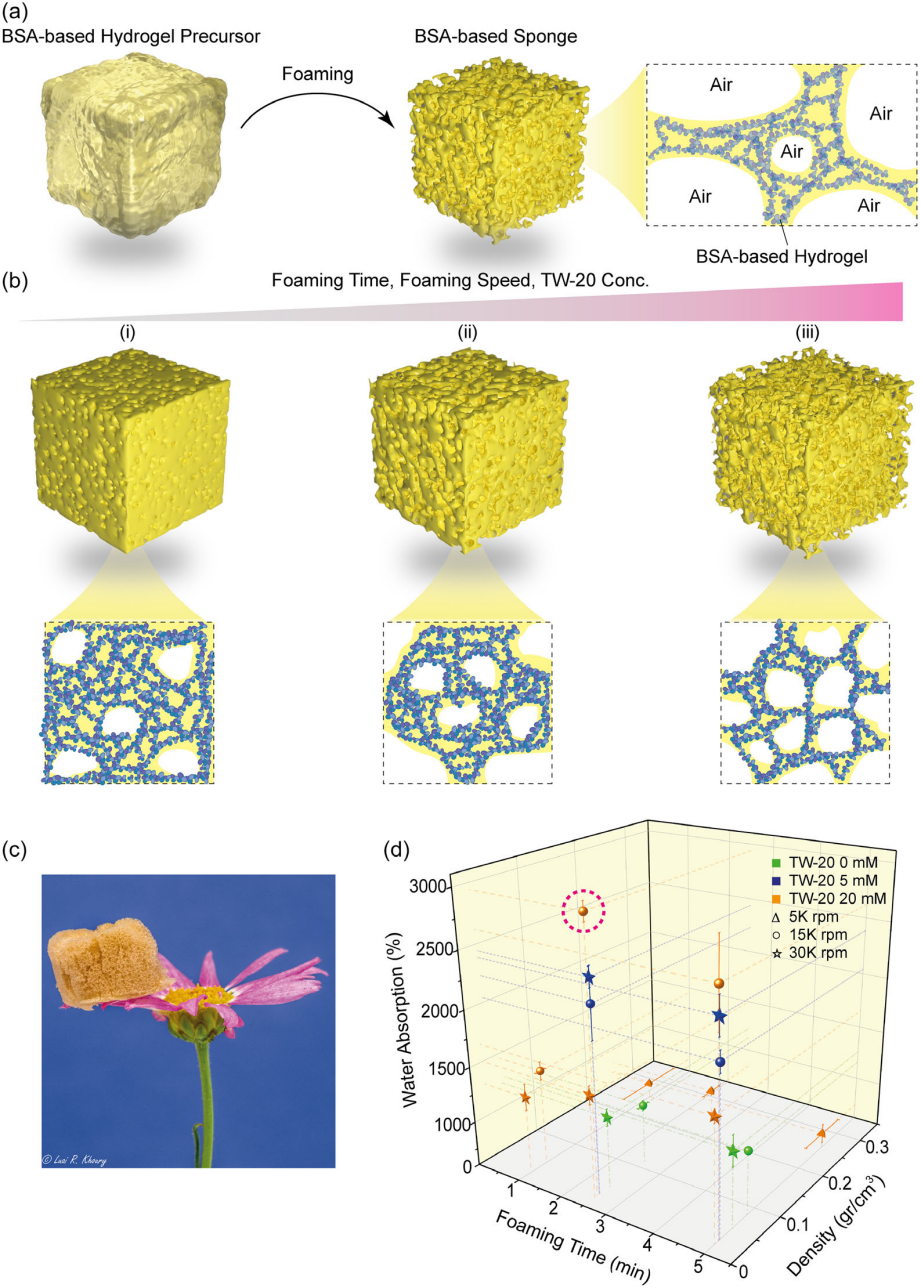
Synthesis, structural stabilization, and water absorption of BSA‐based sponges. a) Schematic of the BSA‐based sponge synthesis process: The process begins with a hydrogel precursor composed of BSA, TW‐20, APS, and Ru (II)bpy_3_^+2^. Homogenization incorporates air, inducing foam formation. The foam is then exposed to white light for 30 min at RT, triggering covalent crosslinking via Ru (II)bpy_3_^+2^ photolysis in the presence of APS. This reaction forms covalent bonds between adjacent tyrosine residues on BSA, yielding a porous sponge network. Inset: Zoom‐in of the BSA‐based sponge structure. b) Effect of foaming parameters on sponge structure: Foaming time, speed, and TW‐20 concentration significantly influence the microstructure and porosity of BSA‐based sponges. (i) Short time, low speed, and  TW‐20 concentration result in a dense hydrogel. (ii) Moderate speed and TW‐20 concentration produce a more open, interconnected porous hydrogel network. (iii) High‐speed and TW‐20 concentration leads to a highly porous sponge. c) Close‐up image of a porous, lightweight BSA‐based sponge after freeze‐drying, placed on a flower petal. d) Density and water absorption measurements for BSA‐based sponges synthesized with varying TW‐20 concentrations (0, 5, and 20 mM) and mixing speeds (5, 15, and 30 K rpm) over different foaming durations (1, 2.5, and 5 min). Sponges prepared with 20 mM TW‐20 at 15 K rpm exhibited the highest water absorption and lowest density (highlighted by the pink dashed circle), indicating optimal sponge formation conditions.

The sponge synthesis began with mixing TW‐20, BSA, Ru (II)bpy_3_^+2^, and APS, resulting in a solution with a final pH of 7.4, followed by foam production using a homogenizer. The foamed mixture was then transferred into cylindrical molds (8 mm × 4.5 mm, Ø  × H) and exposed to white light for 30 min at room temperature (RT), initiating a photoactivated covalent crosslinking reaction between neighboring tyrosine residues on BSA.^[^ ^71^, ^74^, ^75^, ^76^ ^]^ The formed sponges were then washed with a Tris(hydroxymethyl)aminomethane (TRIS) buffer (pH ≈7.4) to remove residual reagents (**Figure** **1**a–c).

To study the influence of foaming parameters on the sponges’ microstructure, we systematically varied mixing speeds (5, 15, and 30 K rpm), durations (1, 2.5, and 5 min), and TW‐20 concentrations (0, 5, and 20 mM), using a fixed BSA concentration of 2 mM.^[^ ^56^, ^77^ ^]^ At a mixing speed of 5 K rpm for 1 min, with 20 mM TW‐20, the resulting hydrogels were opaque, with a density of 0.250 ± 0.018 g cm^−3^ and moderate water absorption (830 ± 38%), suggesting insufficient air entrapment at this lower mixing speed (Figure 1d; Figure S1a, Supporting Information).

Increasing the mixing speed to 15 K rpm improved foam formation. However, a short mixing duration of 1 min caused a gel layer to form at the bottom, yielding sponges with a density of 0.047 ± 0.008 g cm^−3^ and relatively low water absorption (1453 ± 81%). Extending the mixing duration to 2.5 min resolved this issue, producing sponges with stable porous structures, a lower density of 0.0250 ± 0.0003 g cm^−3^, and significantly enhanced water absorption (2796 ± 239%). Further extending the mixing to 5 min did not provide additional benefits, as sponges showed similar densities (0.026 ± 0.001 g cm^−3^) and slightly reduced water absorption (2619 ± 350%) (Figure 1d; Figure S1a, Supporting Information).

At a reduced TW‐20 concentration of 5 mM and a mixing speed of 15 K rpm for 2.5 min, the foam was unstable. Increasing the speed to 30 K rpm improved foam stability, producing sponges with favorable low density (0.028 ± 0.002 g cm^−3^) and high‐water absorption (2440 ± 84%). However, none of the tested conditions produced consistent structures at 5 mM TW‐20 (Figure S1b, Supporting Information). Interestingly, without TW‐20, sponges formed at 30 K rpm had an optimal low density (0.0478 ± 0.0002 g cm^−3^) and water absorption of 1224 ± 74% (Figure 1d; Figure S1c, Supporting Information).

Our results indicate that after 2.5 min of mixing, further increases in foaming duration do not significantly affect sponge density or water absorption. This suggests that the optimal foam structure is achieved within this time frame, regardless of the subsequent mixing time (Figure 1d).

To better understand the influence of foaming parameters on the composition and physical properties of our BSA‐based sponges, we used microCT imaging to visualize their macrostructure (Video S1, Supporting Information). This is the first application of microCT to protein‐based sponges, allowing us to examine their architecture in detail. The nondestructive nature of microCT enabled us to analyze crucial aspects of sponge morphology, such as porosity, density, voids, and fiber orientation, with high precision.^[^ ^78^, ^79^ ^]^ Additionally, the volumetric analysis provided by microCT allowed us to classify the sponges as an open‐pore system, highlighting their interconnected pore network, which is a key feature in ensuring fluid permeability and interaction within the sponge matrix.

**Figure 2:**
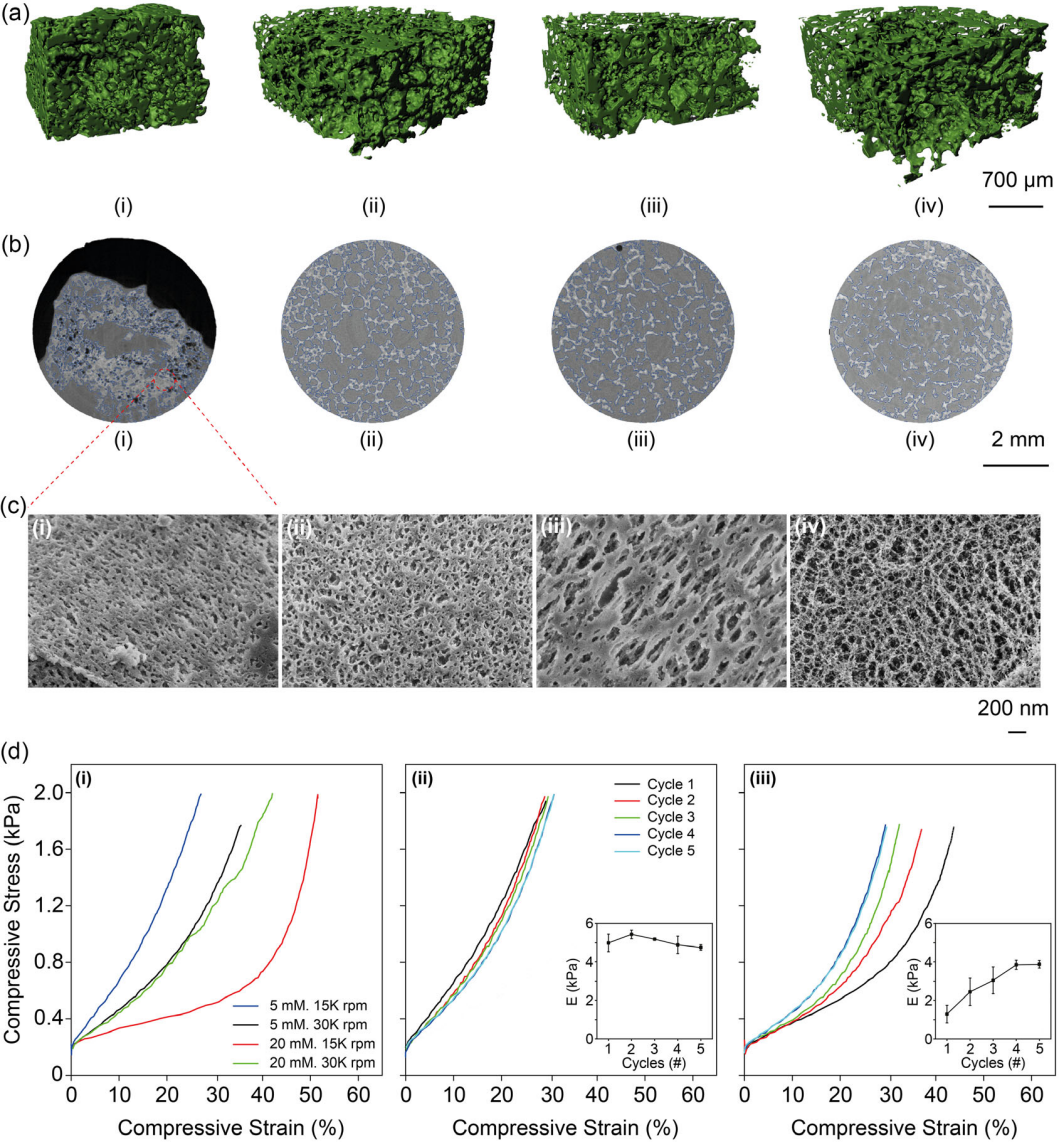
Multiscale morphological and mechanical analysis of BSA‐based sponges under varied foaming conditions. a) The 3D reconstructed microCT scans reveal the internal porosity and structural variations of BSA‐based sponges prepared under different foaming conditions, highlighting the complexity and heterogeneity in pore distribution across samples. b) Cross‐sectional CT images provide a detailed view of BSA distribution, with distinctive blue outlines marking areas enriched in BSA within the sponge matrix, showcasing structural differences between samples. c) Cryo‐SEM images illustrate the microstructure of the hydrogel struts: (i) Sponges without TW‐20, mixed at 15 K rpm for 2.5 min, exhibit densely packed structures with minimal porosity. (ii) Increasing TW‐20 concentration to 5 mM at the same mixing speed and duration enhances porosity, though the BSA network shows nonuniform integration, resulting in uneven pore distribution. (iii) Optimal pore formation and uniform hydrogel strut distribution are achieved with 20 mM TW‐20 and 15 K rpm mixing, showing clearly defined pores and well‐dispersed BSA. (iv) At 30 K rpm and 20 mM TW‐20, the structural integrity of the sponge is compromised, with disrupted hydrogel networks and decreased uniformity. d) Compressive stress–strain analysis: (i) Stress–strain curves of sponges prepared with 5 mM and 20 mM TW‐20 at 15 K and 30 K rpm, showing the effect of foaming conditions on mechanical behavior. (ii) Successive compressive tests for sponges with 5 mM TW‐20 at 15 K rpm reveal stable Young's modulus values (≈5 kPa) across five test cycles. (iii) Successive compressive tests for sponges with 20 mM TW‐20 at 15 K rpm show increasing stiffness over five cycles, with Young's modulus rising from ≈1 to ≈4 kPa, reflecting structural compaction and loss of porosity. The insets show Young's modulus progression across the test cycles.

The 3D microCT images highlighted the significant impact of TW‐20 concentration and foaming speed on sponge morphology. At 15 K rpm without TW‐20, the sponges exhibited a dense structure with 51 ± 2% porosity, indicating a compact hydrogel distribution (**Figure** **2**a,b(i); Figure S2, Supporting Information). Adding 5 mM TW‐20 at the same speed increased porosity but also revealed challenges in achieving an even hydrogel distribution (Figure 2a,b(ii)). Increasing TW‐20 to 20 mM improved porosity to about 72 ± 2%, resulting in better‐defined pores (Figure 2a,b(iii); Figure S2, Supporting Information). However, further increasing the foaming speed to 30 K rpm with 20 mM TW‐20 reduced porosity to 70 ± 8%, demonstrating the complex relationship between foaming dynamics and sponge structure (Figure 2a,b(iv); Figure S2, Supporting Information). This analysis highlights the critical role of microCT in characterizing and optimizing sponge architecture.

Cryo‐SEM was used to further investigate the hydrogel struts’ microstructure. Sponges mixed at 15 K rpm without TW‐20 exhibited a tightly packed microstructure (Figure 2c(i)). Adding 5 mM TW‐20 resulted in a more open, yet uneven, structure (Figure 2c(ii)). At 20 mM TW‐20, a uniform porous structure with an interconnected matrix was observed (Figure 2c(iii)). However, increasing the speed to 30 K rpm at 20 mM TW‐20 led to disorganization and thinner fibrous structures, disrupting the protein network (Figure 2c(iv)).

Porosimetry tests supported the latter findings. Using the Brunauer–Emmett–Teller (BET) model, we determined the surface area of sponges at different foaming speeds. At 20 mM TW‐20, sponges produced at 15 K rpm had a surface area of 1.1562 m^2^ g^−1^, which increased significantly to 4.4772 m^2^ g^−1^ at 30 K rpm. In contrast, sponges with 5 mM TW‐20 exhibited much smaller surface areas—0.0055 m^2^ g^−1^ at 15 K rpm and 1.171 m^2^ g^−1^ at 30 K rpm (Figure S3, Supporting Information). This shows that higher TW‐20 concentrations lead to more consistent and well‐defined pore structures. The Horvath–Kawazoe (HK) model confirmed significant micropore volumes at 20 mM TW‐20 for both 15 and 30 K rpm, with a peak around 12 Å pore width. Lower TW‐20 concentrations showed minimal micropore formation (Figure S4, Supporting Information). The Barrett–Joyner–Halenda (BJH) model indicated the presence of mesopores only at 20 mM TW‐20 for both 15 and 30 K rpm, aligning with the increased porosity observed in the microCT scans (Figure S5, Supporting Information). This confirms TW‐20's role beyond foaming, as it stabilizes the mesoporous structure within the sponge matrix.

To assess how foaming conditions affected mechanical properties, we conducted compressive mechanical tests. Using a force ramp method, we increased the force up to 0.1 N over 60 s to evaluate the sponges’ compressive modulus from the linear region of the stress–strain curve.^[^ ^80^, ^81^ ^]^ This approach provided insights into the material's deformation behavior and structural integrity.^[^ ^82^, ^83^ ^]^ Sponges with 5 mM TW‐20 at 15 K rpm showed higher compressive stress and rigidity, reaching a maximum compressive strain of around 20 ± 2% (Figure 2d(i)). Successive tests revealed that these sponges maintained a stable Young's modulus, starting at 4.9 ± 0.4 kPa in the first cycle and slightly decreasing to 4.7 ± 0.4 kPa in the fifth cycle (Figure 2d(ii)).

In contrast, sponges with 20 mM TW‐20 at 15 K rpm exhibited greater flexibility, reaching a compressive strain of about 50 ± 6% (Figure 2d(i)). With repeated compressive loading, their stiffness increased, with Young's modulus rising from 1.2 ± 0.4 kPa in the first cycle to 3.8 ± 0.1 kPa in the fifth cycle (Figure 2d(iii)). This behavior aligns with the structural characterization, where higher TW‐20 concentrations led to more porous, flexible structures that became stiffer upon successive compressive loading due to pore collapse. In contrast, sponges with lower TW‐20 concentrations were initially denser and maintained their mechanical strength throughout the tests.

At 30 K rpm, sponges with 5 mM TW‐20 exhibited reduced compressive stress and increased compressive behavior, with a compressive strain of 30 ± 3%, indicating a less rigid structure. Sponges with 20 mM TW‐20 at 30 K rpm showed reduced flexibility, with a maximum compressive strain of 40 ± 0.5%, and exhibited fluctuations in their stress–strain curve, suggesting structural weaknesses due to the rapid foaming process (Figure 2d(i)). This analysis underscores the significant impact of foaming conditions on the mechanical properties of BSA‐based sponges, highlighting their potential for applications requiring specific mechanical durability and flexibility.

### 2.2 Evaluating the Structural Integrity of BSA and Adsorption Efficiency of BSA‐Based Sponges for PFOS Removal

**Figure 3:**
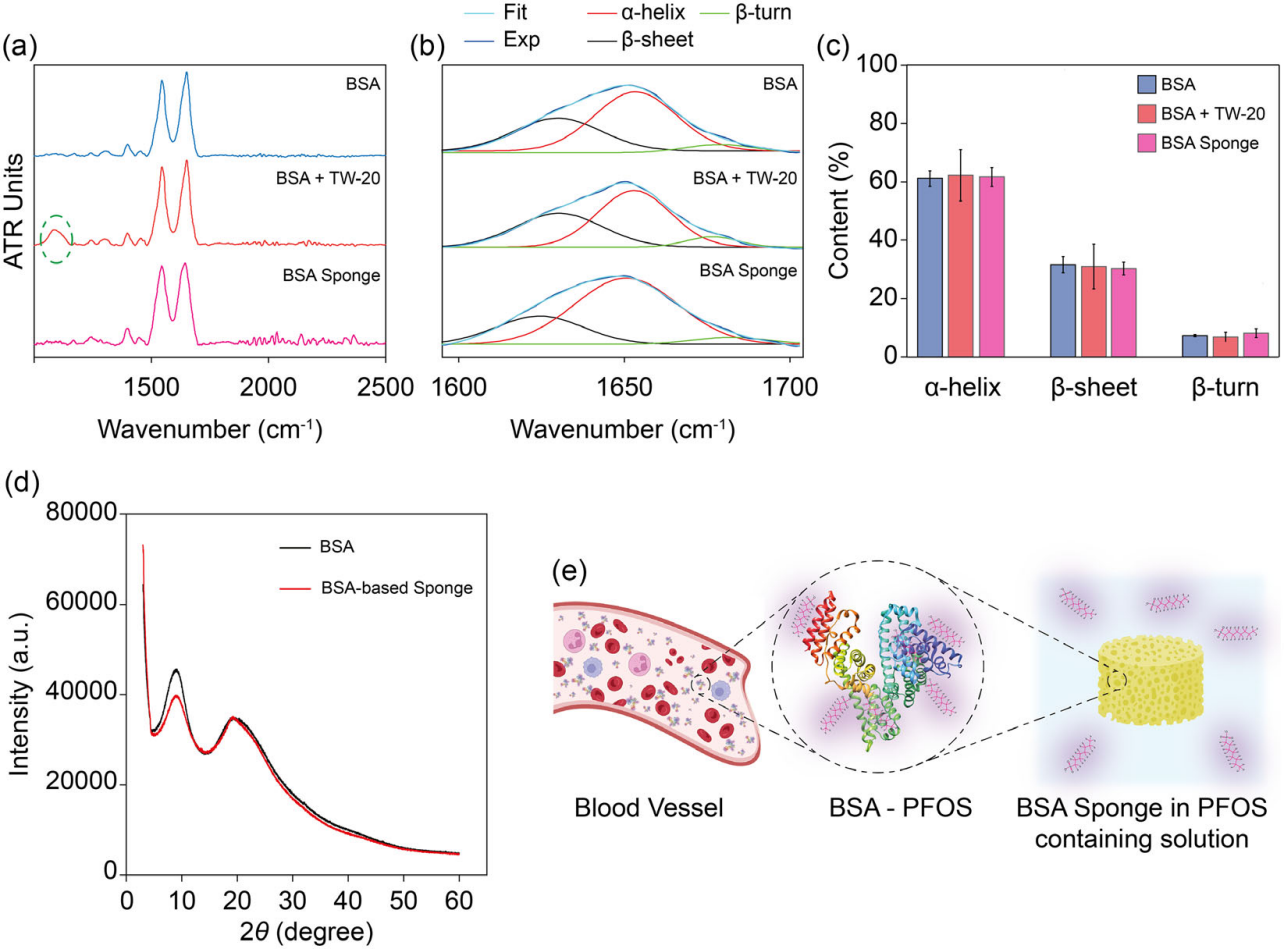
BSA secondary structure preservation and scheme of BSA‐PFOS interactions in BSA‐based sponges. a) ATR‐FTIR spectra of 2 mM BSA in TRIS , 2 mM BSA with 20 mM TW‐20 mixture, and a 2 mM BSA‐based sponge prepared with 20 mM TW‐20. The spectra show effective removal of TW‐20 after washing the sponge with TRIS, indicated by the green‐dashed circle. b) Fourier deconvolution of the amide I band in the ATR‐FTIR spectra is used to analyze the secondary structures of BSA. The deconvoluted spectra reveal the preservation of BSA's key secondary structures—intramolecular β‐sheets, α‐helices, and β‐turns—throughout the sponge formation process. c) The analysis of the amide I band shows that BSA's native secondary structure remains intact across all conditions, demonstrating that neither TW‐20 nor the sponge preparation process significantly alters the protein's conformation before and after gelation. d) XRD patterns of native BSA powder and BSA‐based sponges’ powder. Native BSA exhibited characteristic peaks at 8.94° (*θ*_1_) and 19.30° (*θ*_2_), while the sponges showed corresponding peaks at 9.14° ± 0.03° (*θ*_1_) and 19.20° ± 0.24° (*θ*_2_). The first peak corresponds to ordered regions, potentially α‐helices and β‐sheets, while the second peak reflects amorphous regions. The similarity between the patterns confirms the preservation of BSA's semicrystalline structure during sponge fabrication. e) Schematic representation of serum albumin acting as a PFOS carrier in blood plasma. Inset: The formation of a BSA‐PFOS complex via hydrophobic interactions, electrostatic forces, and hydrogen bonding between BSA amino acids and PFOS. This natural affinity was leveraged in designing BSA‐based sponges to effectively adsorb PFOS for environmental remediation.

After optimizing the fabrication of our BSA‐based sponges, we used ATR‐FTIR to assess the impact of the synthesis process on BSA's secondary structure. This evaluation was critical to ensure that the BSA retained its functionality and structural integrity within the sponge matrix before and after formation. In the ATR‐FTIR spectra, we identified two prominent BSA bands: amide I (1600–1700 cm^−1^) and amide II (1500–1600 cm^−1^) (**Figure** **3**a). The amide I region is particularly sensitive to changes in BSA's secondary structure, as it reflects the conformation of the protein's backbone.^[^ ^84^ ^]^ Therefore, we performed deconvolution analysis of the amide I band for a 2 mM BSA solution without TW‐20, a 2 mM BSA solution with 20 mM TW‐20, and the BSA‐based sponge (20 mM TW‐20, 15 K rpm, 2.5 min).

Our analysis revealed that BSA's secondary structures consist of three primary conformations: intramolecular β‐sheet (1610–1630 cm^−1^), α‐helix (1648–1660 cm^−1^), and β‐turn (1660–1689 cm^−1^) (Figure 3b). The calculated areas under the curves indicated that BSA is composed of ≈30% intramolecular β‐sheet, ≈60% α‐helix, and ≈10% β‐turn, consistent with previous studies.^[^ ^85^, ^86^, ^87^ ^]^ Notably, the BSA's secondary structure remained largely unchanged across different conditions, both in the presence and absence of TW‐20, confirming that the protein's structural integrity is preserved throughout the sponge fabrication process (Figure 3c). To support these findings, XRD was conducted. The XRD patterns revealed two characteristic peaks corresponding to diffraction angles, with 2*θ* values of 9.14° ± 0.03° and 19.20° ± 0.24° for the BSA‐based sponge, closely matching the peaks observed for native BSA at 8.94° and 19.30°,^[^ ^88^, ^89^ ^]^ respectively (Figure 3d). The first peak corresponds to ordered regions, potentially arising from periodic arrangements of α‐helices or β‐sheets, while the second peak reflects the amorphous or disordered regions of BSA, such as loosely packed β‐sheets.^[^ ^90^, ^91^ ^]^ These findings indicate that the semicrystalline nature of BSA, is preserved within the sponge matrix. This preservation is likely due to the weak electrostatic and hydrophobic interactions between TW‐20 and BSA.^[^ ^92^ ^]^ Additionally, the peak observed around 1094 cm^−1^, highlighted by the green circle in Figure 3a, confirms the presence of TW‐20. However, its disappearance after washing the sponge with TRIS buffer further indicates the weak, nonpermanent interaction between BSA and TW‐20 (Figure 3a).

Furthermore, the sponges demonstrated stable composition, with no notable BSA dissociation detected from the sponge matrix (Figure S6, Supporting Information). To validate their environmental safety, we conducted extended leaching experiments to evaluate the potential release of BSA. Bicinchoninic acid (BCA) assay results demonstrated minimal leaching over 10 days, with the cumulative release of BSA reaching only 0.55 ± 0.14% by Day 5 and 1.16 ± 0.14% by Day 10 (Figure S7, Supporting Information). This confirms that the sponges retain their structural integrity under prolonged exposure, underscoring their suitability as environmentally friendly materials with negligible risks of adverse effects. The controlled crosslinking in the BSA‐based sponges significantly stabilizes the matrix, minimizing the release of larger protein fragments and ensuring long‐term structural durability.^[^ ^93^, ^94^ ^]^ This stability suggests that our BSA‐based sponges maintain their structure and functionality, positioning them as promising candidates for various applications.

Given serum albumin's natural tendency to bind PFOS through hydrophobic and other non‐covalent interactions, it is plausible that our BSA‐based sponges will efficiently adsorb PFOS, mimicking the accumulation observed in the human body (Figure 3e).^[^ ^13^ ^]^ The numerous active sites provided by the amino acids in the sponge matrix offer diverse interaction pathways for PFOS adsorption,^[^ ^68^, ^70^, ^95^, ^96^ ^]^ making these BSA‐based sponges a novel and effective tool for targeted PFOS removal (Figure 3e).

XPS analysis was performed to identify functional groups and elucidate the interaction mechanisms between BSA and PFOS by examining binding energy shifts in the C1s, N1s, O1s, S2p, and F1s regions. The results reveal chemical environment changes before and after PFOS exposure (Figure S10 and S11, Supporting Information). In BSA‐based sponge, the C1s spectrum exhibited peaks corresponding to aliphatic and aromatic carbon bonds, amines, and carboxyls. After PFOS exposure on the BSA‐based sponge, new peaks at 291.5 and 293.8 eV, attributed to PFOS's CF and CF_3_ groups, indicated hydrophobic interactions. The N1s and O1s spectra showed shifts consistent with electrostatic and hydrogen bonding between BSA's amine and hydroxyl groups and PFOS's sulfonate groups. The S2p spectrum confirmed sulfur interactions and the presence of PFOS's sulfonate group. Significantly, the superposition of XPS spectra (Figure S12, Supporting Information) demonstrated binding energy shifts, confirming these interactions. Shifts in the C1s region for C—C bonds suggested hydrophobic interactions, while alterations in N1s and O1s indicated electrostatic and hydrogen bonding. The appearance of sulfonate peaks in the S2p region further reinforced the molecular mechanisms underlying PFOS adsorption onto BSA.

Building on the XPS observations, we employed Langmuir and Freundlich isotherm models, along with PFOS detection via liquid chromatography‐tandem mass spectrometry (LC‐MS/MS), to further assess the adsorption processes. The experiments were conducted at pH ≈7.4 using TRIS, simulating the pH of water bodies such as drinking water and rivers where PFOS contamination is prevalent.^[^ ^21^, ^22^, ^23^ ^]^

The Langmuir model, which assumes monolayer adsorption on a homogeneous surface, provided insights into the maximum adsorption capacity (*Q*_max_) and the site affinity constant (*K*_L_).^[^ ^97^ ^]^ In contrast, the Freundlich model accounts for adsorption on heterogeneous surfaces, characterized by constants *K*_f_ and *n*, which represent adsorption capacity and intensity, respectively.^[^ ^98^ ^]^

For this study, BSA‐based sponges (20 mM TW‐20, 15 K rpm, 2.5 min) were selected due to their robust structure, high porosity, and superior water absorption properties (Figure 1 and 2; Figure S2, Supporting Information). PFOS solutions were prepared in ddH_2_O and diluted with TRIS buffer to achieve concentrations ranging from 0 to 1000 ppb. The sponges were immersed in these solutions for varying durations: 20 min, 6 h, and 12 h (**Figure** **4**a(i)). Afterward, the equilibrium adsorption capacity (*q*_e_) was measured, using LC‐MS/MS, based on the equilibrium concentration (*C*_e_) of PFOS after each time interval (Figure 4b; Figure S8, Supporting Information). The highest *q*_e_ was observed at 12 h, reaching ≈7.4 ± 1.0 μg g^−1^ at an equilibrium concentration of ≈440 ppb, while a 20‐min soak resulted in a lower *q*_e_ of 1.5 ± 0.2 μg g^−1^ (Figure 4b).

Figure 4BSA‐based sponges for PFOS Removal: Adsorption mechanism, Efficiency, and Reusability. a) A scheme of (i) BSA‐based sponges immersed in PFOS solutions prepared at varying concentrations. (ii) A passthrough experiment where PFOS solution diluted in TRIS is passed through a sponge‐filled column to assess sponge PFOS removal efficiency. The PFOS removal was assessed using LC‐MS/MS. b) Adsorption isotherm study showing PFOS adsorption on BSA‐based sponges at three soaking times: 20 min, 6 h, and 12 h. The curves show that extended soaking times lead to significantly higher equilibrium adsorption capacities. This suggests that longer exposure allows more PFOS molecules to interact with available binding sites on the sponge surface, contributing to a higher adsorption capacity. c) Adsorption Isotherm Modeling and Data Fitting: (i) Langmuir Isotherm Model – Equilibrium adsorption data fitted to the Langmuir model, assuming monolayer adsorption on a homogeneous surface. The linear fit confirms the model's suitability, indicating that PFOS adsorption onto BSA‐based sponges predominantly follows a monolayer mechanism. Higher equilibrium adsorption capacities at extended soaking times suggest increased PFOS interaction with available binding sites. (ii) Freundlich Isotherm Model – Equilibrium adsorption data fitted to the Freundlich model, which describes multilayer adsorption on heterogeneous surfaces. The linear relationships observed indicate that PFOS initially adsorbs onto binding sites of varying affinities before progressing toward a more uniform distribution over time. The strong fits to both models highlight the complementary nature of the adsorption process, with early‐stage heterogeneity transitioning into monolayer adsorption at longer exposure durations.   d) Time‐dependent PFOS removal by BSA‐based sponges (20 mM TW‐20, 15 K rpm), evaluated after 20 min, 6 h, and 12 h of immersion. At lower concentrations (60 ppb), the sponges exhibited rapid and high adsorption, achieving ≈80% PFOS removal within 20 min. Despite a slight decline in removal efficiency as the PFOS concentration increased, the sponges continued to show robust adsorption, maintaining ≈80% removal at 60 ppb and ≈60% at 1000 ppb, even after 12 h. e) PFOS removal efficiency (%) of BSA‐based sponges over six reuse cycles. The sponges exhibited high initial removal efficiency, with values of ≈74% in the first cycle and ≈68% by the third cycle. This gradually declined to ≈24% in the fifth cycle and ≈10% in the sixth. The decline in PFOS removal efficiency is attributed to the progressive exposure to alkaline pH during washing, which disrupt the protein's native conformation and prevent the complete restoration of its original secondary structure. f) Comparative PFOS removal efficiency of sponges prepared with 5 and 20 mM TW‐20, tested with a 1000 ppb PFOS solution over multiple pass‐through cycles (one, three, and six). The sponges prepared with 20 mM TW‐20 exhibited consistently higher removal rates compared to those with 5 mM TW‐20, with the difference becoming more pronounced as the number of cycles increased. After six cycles, sponges with 20 mM TW‐20 reached a removal efficiency of ≈45%, while sponges with 5 mM TW‐20 achieved ≈36%. A column packed with three 20 mM TW‐20 sponges achieved ≈89% PFOS removal, attributed to increased surface area and interaction time. g) Comparative radar chart analysis of BSA‐based sponges versus other PFOS removal methods, including magnetic activated carbon (MAC), powder activated carbon (PAC), anion‐exchange resins, and electrocoagulation. The chart highlights the superior biocompatibility, microstructure controllability, and competitive PFOS removal efficiency of BSA‐based sponges, along with advantages in cost and synthesis complexity over traditional methods.

**Figure:**
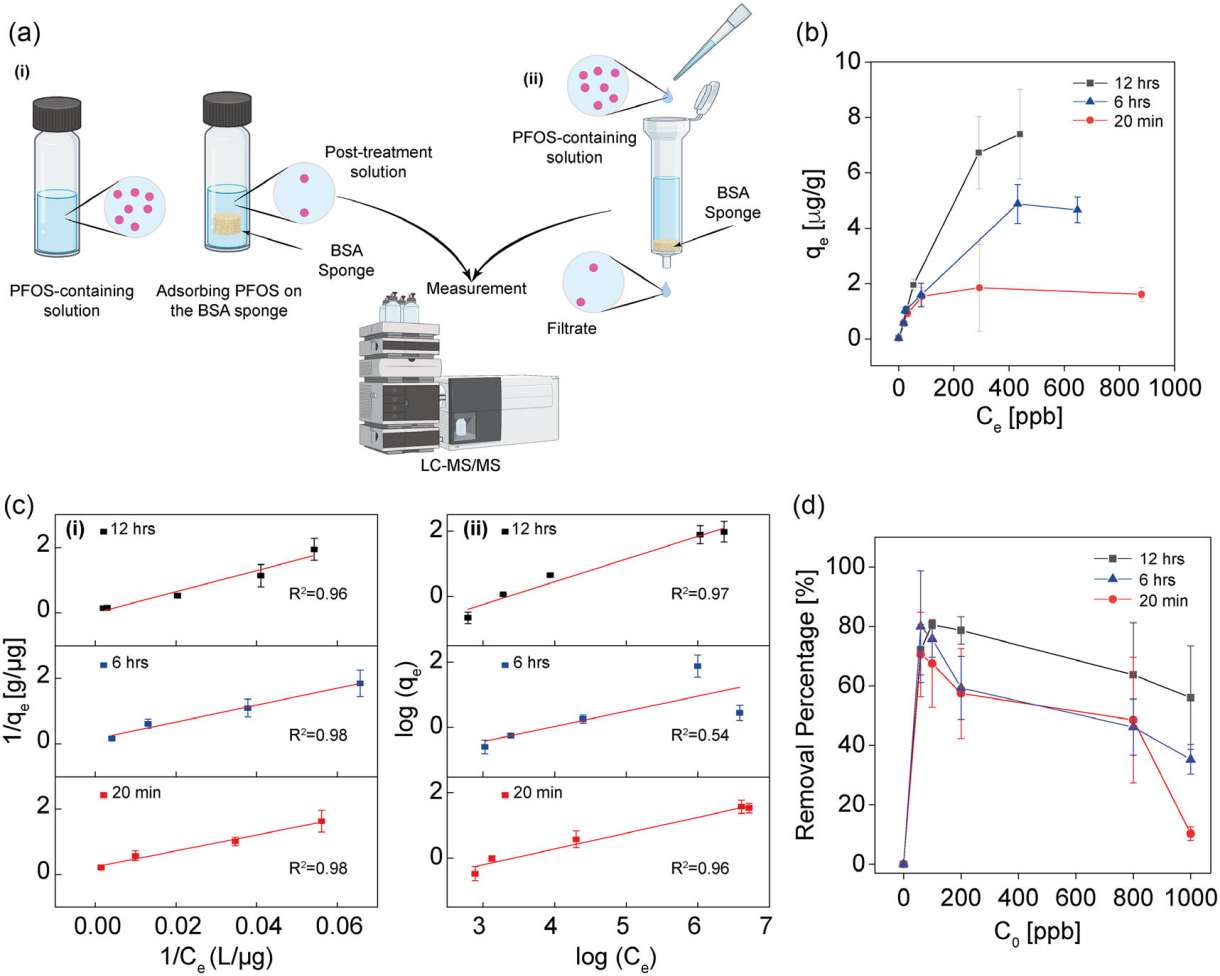

**Figure:**
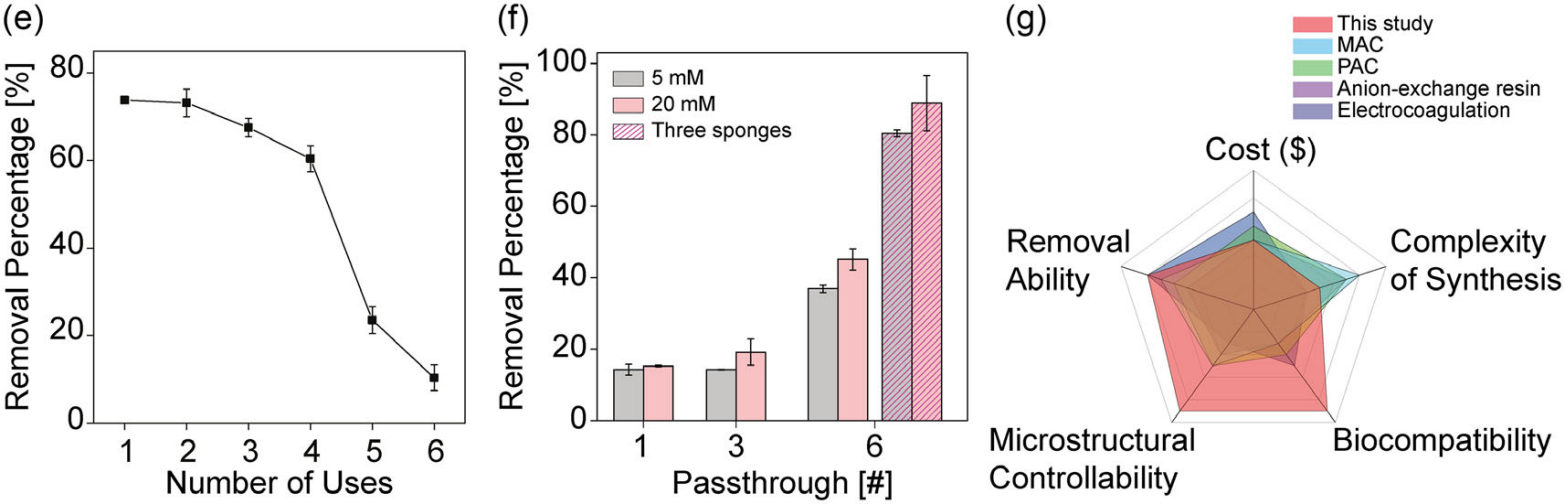

Fitting the data to both the Langmuir and Freundlich models provided complementary insights. The Langmuir model exhibited an excellent fit, suggesting predominant monolayer adsorption on a homogenous surface. The maximum adsorption capacities (*Q*_max_) were 4.7 ± 1.6 μg g^−1^, 6.8 ± 0.1 μg g^−1^, and 303.0 ± 20.3 μg g^−1^ for soaking times of 20 min, 6 h, and 12 h, respectively. A decline in the Langmuir constant (*K*_L_) over time indicated reduced site affinity as more sites became occupied (Figure 4c(i) and **Table** **1**).

**Table 1:** Parameters of Langmuir and Freundlich adsorption isotherm models for PFOS adsorbed by BSA‐based sponges.

Adsorption duration	Langmuir model			Freundlich model		
	$Q_{\text{max}}$ $\left[\frac{\mu g}{g}\right]$	$K_{L}$ $\left[\frac{L}{\mu g}\right]$	$R^{2}$	*n*	$K_{f}$ $[\frac{\mu g}{g}{\left(\frac{L}{\mu g}\right)}^{\frac{1}{n}}]$	$R^{2}$
20 min	4.7 ± 1.6	9.0 × 10^−3^ ±8.1 × 10^−4^	0.98 ± 0.19	1.9 ± 0.7	1.9 × 10^−2^ ±9.6 × 10^−3^	0.96 ± 0.06
6 h	6.8 ± 0.1	4.8 × 10^−3^ ±8.2 × 10^−4^	0.98 ± 0.12	2.3 ± 0.7	2.1 × 10^−2^ ±5.6 × 10^−3^	0.54 ± 0.05
12 h	303.0 ± 20.3	7.4 × 10^−5^ ±1.3 × 10^−5^	0.96 ± 0.09	1.4 ± 0.04	4.9 × 10^−3^ ±1.0 × 10^−3^	0.97 ± 0.01

The Freundlich model, particularly relevant at shorter soaking times, effectively captures the adsorption behavior on a heterogeneous surface with variable site energies. This is especially relevant given the intrinsic structural and chemical heterogeneity of BSA‐based sponges. Their hierarchical structure, characterized by α‐helices, β‐sheets, and β‐turns, generates diverse surface features, while the porous network formed during the foaming process with TW‐20 introduces variations in pore size, surface roughness, and pore wall composition.^[^ ^99^, ^100^ ^]^ These structural and chemical variations result in a nonuniform distribution of active binding sites, influencing adsorption energies and reinforcing the suitability of the Freundlich model to describe such complex interactions.^[^ ^101^, ^102^, ^103^ ^]^ This observation aligns with the 1/*n* values, which were less than 1 in all cases, indicating favorable adsorption conditions. Additionally, the *K*_f_ values further highlighted the sponge's strong adsorption capacity, especially at lower PFOS concentrations, demonstrating its efficiency during the initial adsorption phase. The strong correlation coefficients (*R* ^2^ ≈1) across these intervals further supported the model's applicability (Figure 4c(ii) and Table 1).

After determining the equilibrium adsorption capacity and evaluating the adsorption models, the removal percentage of PFOS by the sponges was calculated by determining the difference between the initial PFOS concentration and the equilibrium concentration after adsorption. In the initial 20 min, the sponges demonstrated high removal rates, achieving around 80 ± 14% efficiency at 60 ppb. This effectiveness persisted at higher PFOS concentrations and longer soaking times, with consistent removal rates observed at concentrations of 200 and 800 ppb over 6 h. After 12 h, the sponges continued to show high performance, maintaining around 80 ± 1% removal for concentrations from 60 to 200 ppb. However, a decline in removal efficiency was observed at concentrations of 800 and 1000 ppb, with a decrease to ≈60 ± 17% (Figure 4d). This behavior is consistent with adsorption models like Langmuir, where a maximum adsorption capacity is eventually reached as concentration increases.

To evaluate the reusability of BSA‐based sponges, adsorption–desorption experiments were performed over six cycles. The sponges were immersed in an 800 ppb PFOS solution for 12 h, followed by a 2 h washing step in TRIS buffer (pH ≈10) under rotational mixing to disrupt noncovalent interactions such as hydrogen bonding and electrostatic forces.^[^ ^104^, ^105^ ^]^ This alkaline washing induced changes in the sponge's secondary structure, with ATR‐FTIR analysis showing an increase in β‐sheet content to 49.6 ± 8.6% and a reduction in α‐helix content to 40.3 ± 0.7% (Figure S9, Supporting Information). To neutralize residual alkaline solution and restore structural integrity, the sponges were washed for 2 h with TRIS buffer (pH ≈7.4) (Figure S9, Supporting Information). This process was repeated for six cycles, with PFOS removal efficiency measured after each cycle using LC‐MS/MS. The results revealed that removal efficiency remained high during the first three cycles, starting at 73.8 ± 0.7% in the first cycle and decreasing to 67.6 ± 2.1% by the third cycle. However, a significant decline occurred from the fourth cycle onward, dropping to 60.4 ± 3.0% in the fourth cycle and further to 10.3 ± 2.9% by the sixth cycle (Figure 4e). This decline is attributed to repeated exposure to alkaline conditions, which disrupt the protein's native conformation. The secondary structure of BSA, which consists of ≈60% α‐helix, ≈30% β‐sheet, and ≈10% β‐turn, is critical for its conformational flexibility and stability in various environments. This structural feature allows BSA to maintain its functional properties, including its ability to bind and adsorb molecules effectively.^[^ ^106^, ^107^ ^]^ Therefore, the reduction in α‐helix content to ≈40% and the increase in β‐structures to ≈60% after repeated cycles may impair the sponge's ability to maintain its adsorption capacity over successive cycles (Figure S9, Supporting Information).

Additionally, pass‐through experiments were conducted to investigate the relationship between the sponge's microstructure—specifically its porosity, surface area, water retention capacity, interaction time, and its PFOS removal efficiency (Figure 4a(ii)). A 1000 ppb PFOS solution was passed through a column containing a single BSA‐based sponge (15 K rpm, 2.5 min) prepared with either 5 or 20 mM TW‐20. The PFOS concentration in the filtrate was measured to assess the sponge's efficiency. Initially, sponges prepared with 20 mM TW‐20 removed about 15 ± 0.2% of PFOS, while those with 5 mM TW‐20 showed a similar uptake of 14 ± 1.5% (Figure 4f).

With repeated pass‐through cycles (three and six cycles), all sponges demonstrated improved PFOS removal. After six cycles, sponges with 20 mM TW‐20 achieved a removal rate of ≈45 ± 3.0%, compared to 36 ± 1.0% for those with 5 mM TW‐20. When three sponges were used in a column for six cycles, the sponges of 20 mM TW‐20 reached a removal efficiency of 89 ± 7.7%, while those with 5 mM TW‐20 showed an efficiency of 80 ± 1.0% (Figure 4f). The superior performance of the 20 mM TW‐20 sponges can be attributed to their optimized microstructure, which provides enhanced porosity and absorption properties (Figure 1d).

In summary, PFOS adsorption onto BSA‐based sponges is well described by both the Langmuir and Freundlich isotherms, each revealing different aspects of the process, underscoring the potential of BSA‐based sponges as effective adsorbents for environmental remediation. The Langmuir model's excellent fit across all soaking times highlights the predominance of monolayer adsorption on a relatively uniform surface. Meanwhile, the Freundlich model suggests that adsorption initially occurs on heterogeneous sites. However, as PFOS concentrations rise, a drop in adsorption efficiency is noted due to the saturation of available binding sites, as explained by the Langmuir model. Thus, while prolonged exposure enhances removal at lower concentrations, efficiency declines at higher levels due to saturation effects.^[^ ^97^, ^108^ ^]^

Protein‐based sponges have gained considerable attention due to their inherent biocompatibility, porous structure, and absorbent nature. However, conventional synthesis methods often involve multiple steps under harsh conditions, which can degrade the protein's structure and functionality. For example, silk fibroin sponges suffer from reduced flexibility and water absorption, which can be partially mitigated by additives like konjac glucomannan, but this compromises control over swelling and structural morphology.^[^ ^109^ ^]^ Similarly, whey protein sponges require hot air and microwave heating during synthesis, risking premature protein denaturation.^[^ ^65^ ^]^

In contrast, our approach advances the field by developing fully functional BSA‐based sponges without synthetic additives to enhance porosity and water absorbency (Figure 1 and 2). This method preserves BSA functionality (Figure 3), avoiding the denaturation often caused by harsh processing conditions. Furthermore, it eliminates the need for drying processes, representing a significant improvement over the previous strategy.

Unlike traditional surfactant‐modified porous materials, which often neglect the impact of foaming speed and duration, our study systematically evaluates these parameters to optimize sponge quality. By exploiting the synergistic interactions between TW‐20 and BSA, we achieved controlled foam formation that balanced macro‐ and microstructural integrity, resulting in consistent material stability. Porosimetry tests confirmed that higher TW‐20 concentrations significantly improved pore structure uniformity and surface area, underscoring the role of surfactant concentration in optimizing sponge porosity. TW‐20 facilitates uniform foam formation via the Gibbs–Marangoni effect,^[^ ^110^, ^111^ ^]^ while BSA enhances film durability due to its viscoelastic gel properties.^[^ ^112^ ^]^ Mechanical tests further demonstrated that optimal foaming conditions increased the sponges’ compressive strength and resilience, showcasing the direct influence of foam dynamics on mechanical properties. Building on these structural and mechanical advantages, the biocompatibility of the materials used in our BSA‐based sponges further underscores their versatility and potential for diverse applications. Key components such as BSA, TW‐20, APS, and Ru(II)bpy_3_^+2^ are well documented for their safe use in biomedical and environmental contexts. For instance, BSA, a major serum protein, is used in biomedical applications such as drug delivery and tissue engineering due to its natural compatibility with biological systems.^[^ ^113^, ^114^, ^115^ ^]^ TW‐20 is a non‐ionic surfactant frequently utilized in pharmaceuticals for its low toxicity profile, while APS, at the controlled concentration used in our study (0.06 M), aligns with established safe ranges for hydrogel formation in biomedical applications.^[^ ^116^, ^117^, ^118^, ^119^ ^]^ Similarly, Ru(II)bpy_3_^+2^, employed as a crosslinking agent, has demonstrated minimal cytotoxicity at the used concentration (0.4 mM), as supported by prior studies.^[^ ^120^, ^121^ ^]^ This approach offers significant advancement in protein‐based sponge synthesis, providing a framework for designing future materials with tailored structural and functional properties for various applications.

Our BSA‐TW‐20 methodology also provides significant advantages over traditional PFOS removal methods such as MAC, PAC, anion‐exchange resins, and electrocoagulation (Figure 4g). For example, while MAC shows a high adsorption capacity (≈666.6 μg g^−1^) in wastewater, its complex synthesis process and limited biocompatibility pose challenges.^[^ ^122^ ^]^ Similarly, ultrafine magnetic PAC, though effective in wastewater treatment with a capacity of 330 μg g^−1^ at low pH, loses efficiency at neutral pH, which is critical for real‐world applications. Its manufacturing process is complex because it requires precise control and multiple steps, ultimately limiting its practical application in large‐scale operations.^[^ ^24^, ^122^ ^]^ Anion‐exchange resins, with an average removal efficiency of 266.5 μg g^−1^, are disadvantaged by high material costs and complex synthesis.^[^ ^123^ ^]^ Electrocoagulation, though effective in generating large flocs for PFOS removal, can produce harmful byproducts and is energy‐intensive, with the added challenge of maintaining electrode functionality.^[^ ^124^ ^]^

Our BSA‐based sponges demonstrate a competitive adsorption capacity of 303.03 μg g^−1^, achieved through a simple and cost‐effective synthesis process‐utilizing natural, biocompatible materials without relying on hazardous chemicals. This method significantly reduces energy consumption and operational costs compared to conventional high‐pressure membrane processes such as reverse osmosis and nanofiltration. These traditional methods typically consume 1–10 kWh m^−3^ of energy, resulting in operational costs of $0.60–$1.50 per cubic meter.^[^ ^125^, ^126^, ^127^, ^128^, ^129^ ^]^ In contrast, our sponge synthesis requires only minimal energy inputs, and the passive nature of PFOS adsorption further enhances its efficiency. This combination of low‐energy requirements and sustainable materials makes our approach a more practical and cost‐effective solution for large‐scale applications.

However, the sponges’ removal efficiency decreases slightly at higher PFOS concentrations (800–1000 ppb), likely due to saturation effects where the available binding sites become limited. This behavior is consistent with the Langmuir adsorption model, which suggests that as solute concentration increases, the adsorbent reaches a maximum capacity. Despite the slight decrease in efficiency at higher concentrations, the sponges maintained strong removal performance even at elevated PFOS levels (Figure 4d). This demonstrates their practical potential, especially since their controlled micro‐ and macrostructure allow for tailored porosity and mechanical properties, enhancing PFOS adsorption. Our method offers a safer, more eco‐friendly approach to PFOS removal compared to traditional methods and lays the groundwork for designing protein‐based sponges tailored for various applications (Video S2, Supporting Information). Inspired by serum albumin's natural affinity for PFOS binding, we leveraged foam stability and protein interactions to create a versatile system. This approach could be adapted to target and remove other hazardous substances, expanding the applications of protein‐based materials for environmental remediation.^[^ ^130^, ^131^ ^]^

While our findings are promising, challenges remain. Future work should focus on improving the reusability of BSA‐based sponges and developing efficient disposal methods. Additionally, exploring surface modifications could help increase PFOS selectivity and reduce nonspecific adsorption.^[^ ^132^ ^]^ These refinements could further enhance the practical applicability of our sponges in environmental cleanup efforts.

## 3 Conclusions

This study demonstrates the successful synthesis of BSA‐based sponges that effectively adsorb PFOS, with TW‐20 playing a critical role in optimizing the foam's macro‐ and microstructure. By fine‐tuning the foaming parameters, we developed a material with enhanced stability, porosity, and functionality, surpassing the performance of traditional surfactant‐modified materials. The preserved structural integrity of BSA, combined with the high affinity of the sponges for PFOS, highlights this method as a promising, biocompatible, and sustainable solution for addressing PFOS contamination in the environment.

Our results show that BSA‐based sponges not only achieve high PFOS removal efficiency but also offer a simpler, more cost‐effective synthesis process using natural materials. The ability to control the sponges’ porosity and mechanical properties further enhances their versatility for a wide range of environmental applications. In addition to their potential for environmental remediation, these sponges’ biocompatibility and structural tunability make them suitable for biotechnological advancements, such as tissue engineering scaffolds, wound healing, drug delivery systems, and biomolecule filtration and sensing platforms.

## 4 Experimental Section

4.1

4.1.1

##### Materials

BSA lyophilized powder, 1× crystallized, ≥97%; polyethylene glycol sorbitan monolaurate (TWEEN 20); APS ACS reagent, ≥98.0%; Tris(2,2′‐bipyridyl)dichlororuthenium(II) hexahydrate (Ru(II)bpy_3_^+2^); Trizma base, ≥99.8%; sodium chloride flakes; heptadecafluorooctanesulfonic acid solution (PFOS), ≈40% in H_2_O; and iodine/potassium iodide solution (Lugol solution) were all purchased from Merck Sigma‐Aldrich.

##### BSA‐based Sponge Synthesis

BSA powder was dissolved in TRIS buffer (20 mM Tris, 150 mM NaCl, pH ≈7.4) containing varying concentrations of TW‐20 surfactant (0 mM, 5 mM, and 20 mM) to achieve a final BSA concentration of 2 mM. The BSA/TW‐20 solution (450 μL) was mixed with Ru (II)bpy_3_^+2^ (6.67 mM, 30 μL) and APS (1 M, 30 μL) in a 15:1:1 volume ratio. The mixture was homogenized using a Bio‐Gen PRO200 homogenizer at controlled foaming speeds of 5, 15, or 30 K rpm for 1, 2.5, or 5 min at RT. After homogenization, the generated foam was transferred into cylindrical molds (8 mm diameter) and exposed to white LED light (1000 lux) for 30 min to induce covalent crosslinking between tyrosine residues on the BSA, thereby forming the sponge structure. Upon completion of the crosslinking process, the sponges were removed from the molds and washed three times with TRIS buffer at RT to eliminate residual TW‐20 and unreacted intermediates. The sponges were then ready for subsequent characterization and application tests.

##### Density Measurements

Foam samples containing 2 mM BSA and varying concentrations of TW‐20 were prepared and washed three times with deionized water (ddH_2_O) at RT to remove residual reagents. The cleaned sponge samples were freeze‐dried using liquid nitrogen (77 K) and subsequently transferred to a lyophilizer under vacuum (0.016 mBar). After 24 h, the dry weight ($W_{\text{dry}}$) of each sponge was measured using an analytical balance. For volume determination, images of the dry sponge samples were captured, and their dimensions were measured using ImageJ software (NIS, USA). The volume (*V*) of each sponge was then calculated based on these measurements. The density (*ρ*) of each sponge was determined using the equation $\rho =W_{\text{dry}}/V$. For sponges prepared with 2 mM BSA and 0 mM TW‐20, precise volume measurements were obtained using a Micromeritics AccuPyc II 1340 Helium Pycnometer (analysis conditions: temperature 19.4 °C and helium pressure 19.5 psi) to ensure accuracy in volume calculation.

##### Water Absorption Measurement

BSA‐based sponges prepared with varying concentrations of TW‐20 were tested in triplicate for water absorption. The sponges were immersed in TRIS buffer for 24 h at 4 °C to ensure complete swelling. After immersion, the sponges were gently blotted with filter paper to remove excess buffer and immediately weighed to record the wet weight ($W_{\text{wet}}$). Following this, the sponges were washed three times with deionized water (ddH_2_O) at RT to remove any residual buffer. The samples were then freeze‐dried and weighed again to determine their dry weight ($W_{\text{dry}}$). The water absorption ratio was calculated using the following equation.

(1) Wwet−WdryWdry*100

##### Porosimetry Tests

The micropore and mesopore size distributions of the BSA‐based sponges were characterized using nitrogen adsorption and desorption measurements at 77 K, performed with a 3Flex Surface Characterization Analyzer (Micromeritics, Norcross). Micropore size distributions were derived from the adsorption curves using the HK model, assuming cylindrical pore geometry. Mesopore size distributions were determined from the adsorption data using the BJH model. Additionally, the specific surface area (S(BET)) was calculated using the multipoint BET model, based on 5‐point adsorption isotherms. These measurements provided a comprehensive assessment of the pore size distribution and surface area of the sponges.

##### Mechanical Characterization

For the compressive tests, cylindrical BSA‐based sponge samples (8 mm in diameter and 4 mm in height) prepared with varying concentrations of TW‐20 were washed and equilibrated in TRIS buffer for 3 h. After equilibration, the dimensions of each sponge were measured using a digital Vernier caliper (NEIKO). Compression tests were performed using a dynamic mechanical analyzer (Anton Paar). The tests followed a ramp linear force profile, collecting 1000 data points at intervals of 0.6 s over a total duration of 60 s. The applied force began at 0.01 N and increased linearly to 0.1 N. The compressive modulus was determined from the linear portion of the resulting stress–strain curves. For successive compressive tests, each sponge was resoaked in TRIS buffer for 1 h to allow for structural recovery. After resoaking, the dimensions were remeasured, and the compressive test was repeated. This cycle of compression, recovery, and remeasurement was performed a total of five times for each sponge sample, ensuring consistent evaluation of mechanical properties across multiple test cycles.

##### Cryo‐SEM

BSA‐based sponges were prepared and washed with deionized water (ddH_2_O) at RT. The washed samples were placed between two aluminum discs (3 mm in diameter, 25 μm thick each) and cryoimmobilized using a high‐pressure freezing device (EM ICE, Leica). The cryoimmobilized samples were mounted on a holder under liquid nitrogen in a loading station (EM VCM, Leica) and transferred under cryogenic conditions using a cryotransfer system (EM VCT500, Leica) to a freeze‐fracture device (EM ACE900, Leica) for sample preparation. The samples were fractured by a rapid stroke of a cryogenically cooled knife to expose the internal structure between the discs. Following fracture, the samples were etched at −100 °C for 10 min to sublimate surface ice and then coated with a 3 nm layer of carbon. Imaging was performed using a secondary electron in‐lens detector with a Gemini SEM (Zeiss), operating at −120 °C. All measurements were conducted at the Ilse Katz Institute for Nanoscale Science and Technology, Ben‐Gurion University of the Negev, Beer Sheva, Israel.

##### ATR‐FTIR

ATR‐FTIR spectra were obtained from both solutions and sponges containing 2 mM BSA with varying TW‐20 concentrations (0 mM and 20 mM) using a Nicolet iS50 FTIR instrument in ATR mode. The spectra were recorded using a round Diamond (Type IIa crystal) ATR accessory. For each sample, 16 scans were collected with a nominal resolution of 8 cm^−1^. The conformational changes in the three primary secondary structures of BSA—namely intramolecular β‐sheets (1610–1630 cm^−1^), α‐helix (1648–1660 cm^−1^), and β‐turns (1660–1689 cm^−1^)—were analyzed by deconvoluting the amide I band (1600–1700 cm^−1^). Spectral deconvolution and analysis were performed using OMNIC FTIR Software to provide insights into the structural integrity of BSA in both solution and sponge states.

##### XRD

BSA‐based sponges were prepared using 20 mM TW‐20 (15 K rpm, 2.5 min). After preparation, the sponges were washed with ddH_2_O and freeze‐dried. The sponges were then ground into a fine powder using a pestle. For comparison, original BSA powder was used directly without any additional processing. The powdered samples were analyzed using a Rigaku SmartLab 9 kW X‐ray diffractometer equipped with a Cu–Kα radiation source. The measurements were carried out with a beam voltage of 45 kV and a beam current of 200 mA, employing parallel beam optics for high‐resolution analysis. The diffraction data were collected over a 2*θ* range of 0° to 60°, with a scan step size of 0.01° and a scan speed of 3° per minute.

##### BCA Assay

BSA‐based sponges prepared with 20 mM TW‐20 (15 K rpm, 2.5 min) were soaked in TRIS buffer (pH ≈7.4) under rotation at room temperature. Samples were collected from the surrounding buffer after 24 h over a 10‐day period for protein content analysis using the BCA assay. The absorbance of each sample was measured at 562 nm using a Tecan Infinite 200 PRO spectrophotometer. A calibration curve was prepared using a series of BSA standard solutions with concentrations of 0, 25, 50, 100, 200, 400, 800, and 1000 μg mL^−1^ to compute the concentration of the released BSA.

The cumulative release of BSA (%) was calculated using the equation.

(2) Cumulative release of BSA (%)=  MtM∞*100

where *M*_*t*_ is the total amount of BSA released at time *t* (in μg) and *M*_∞_ is the total amount of BSA in the sponge (7500 ± 0.0005 μg).

##### MicroCT

BSA‐based sponges were prepared with varying TW‐20 concentrations (0, 5, and 20 mM) and subjected to centrifugation for 1 min at 1500 rpm to eliminate trapped air bubbles. The sponges were then immersed in 5 mL of Lugol solution (diluted I_2_/KI solution at 15% v/v) and stored in the refrigerator at 4°C for 3 days to enhance contrast for microCT imaging. Following the Lugol staining, the sponges were positioned into the caps of Eppendorf tubes and mounted horizontally in a sample holder facing the scanning panel. During the scan, the source voltage was set at 55 kV, and the current at 72 μA. The imaging was performed with a pixel resolution of 10.50 μm, using a 360° rotation with a rotation step of 0.150°. At each angle, three images were captured. Each scan took ≈6 to 8 h per sample, ensuring comprehensive image acquisition with an exposure time of 1150 ms. The raw data obtained from the scans were reconstructed into Z‐stack images, which were processed using the Watershed algorithm to distinguish the pores from the protein hydrogel compartments. A 3D model of each sponge was then generated, and both horizontal and vertical cross‐sectional images were acquired. Porosity calculations were carried out using CTan software (BRUKER). All microCT scans were conducted at the Biomedical Core Facility, Faculty of Medicine, Rambam Medical Center.

##### PFOS Adsorption and Removal by BSA‐based Sponges

BSA‐based sponges (20 mM TW‐20, 15 K rpm, 2.5 min) were immersed in PFOS‐containing solutions for varying durations to evaluate adsorption efficiency. PFOS solutions were prepared by diluting different volumes of a PFOS stock solution (1 mg mL^−1^ in water) into TRIS buffer to obtain final PFOS concentrations of 0, 60, 100, 200, 800, and 1000 ppb in a total volume of 3 mL. The sponges were subjected to rotational immersion in these PFOS solutions for 0, 0.3, 6, and 12 h. After immersion, the remaining PFOS in the solution was first separated using HPLC and then detected and quantified by LC‐MS/MS. To assess the reusability of the sponges, BSA‐based sponges were immersed in 800 ppb PFOS solutions for 12 h under rotational mixing, followed by measurement of PFOS removal using LC‐MS/MS. After the adsorption step, the sponges were subjected for 2 h washing process using TRIS buffer (pH ≈10) under rotational mixing to disrupt noncovalent interactions between PFOS and the BSA‐based sponge matrix. Subsequently, the sponges were washed with TRIS buffer (pH ≈7.4) for an additional 2 h to neutralize and remove residual alkaline solution. This adsorption‐desorption cycle was repeated six times, with PFOS removal efficiency evaluated after each cycle.

The LC‐MS/MS analysis was performed using a Maxis Impact HD Mass Spectrometer equipped with electrospray ionization in positive mode, and a Phenomenex Luna C18(2) column (5 μm, 100 Å pore size, 150 mm × 2.00 mm). Mobile phase A consisted of 2 mM ammonium acetate in water, and mobile phase B was acetonitrile. The gradient elution program for phase B started at 80.0% at 0 min, decreased to 30.0% at 3.0 min, held until 4.0 min, dropped to 0.0% at 5.0 min, and held constant until 8.0 min, before increasing to 65.0% at 9.0 min and held constant until the end of the run at 15.0 min.

Mass spectrometric detection was performed with an end plate offset of 500 V and a capillary voltage of 4000 V for positive ion mode. Nebulizer pressure was set at 3.0 bar, dry gas flow at 8.0 L min^−1^, and dry gas temperature at 200 °C. For negative ion mode, the end plate offset was set to −500 V, and the vaporizer temperature was set to 200 °C. PFOS was consistently detected at a retention time of 4.6 min. PFOS concentrations in the samples (0, 60, 100, 200, 500, and 900 ppb) were quantified using a calibration curve generated by plotting the peak area against known PFOS concentrations.

###### PFOS Adsorption and Removal by BSA‐based Sponges: Adsorption and Removal Calculations

The percentage of PFOS removal was calculated using the formula.

(3) $$\text{Removal Percentage \%}=\frac{C_{0}-C_{e}}{C_{0}}$$

where *C*_0_ is the initial concentration of the solute in the solution (ppb) and *C*_e_ is the equilibrium concentration of the solute in the solution (ppb).

The amount of solute adsorbed per unit weight of the adsorbent (*q*_e_) was then calculated using the equation.

(4) $$q_{e}=\frac{\left(C_{0}-C_{e}\right)\times V}{m}$$

where *q*_e_ is the amount of solute adsorbed per unit mass of adsorbent at equilibrium (μg/g), *C*_0_ is the initial concentration of the solute in the solution (ppb), *C*_e_ is the equilibrium concentration of the solute in the solution (ppb), *V* is the volume of the solution (L), and m is the mass of the adsorbent used (g), with an average value of 0.23 g used in these experiments.

###### PFOS Adsorption and Removal by BSA‐based Sponges: Adsorption Isotherm Models

The adsorption behavior of the BSA‐based sponges was analyzed using both the Langmuir and Freundlich isotherm models. The linear form of the Langmuir isotherm model was given by

(5) $$\frac{1}{q_{e}}=\frac{1}{Q_{\text{max}}c_{e}}+\frac{1}{Q_{\text{max}}K_{L}}$$

where *Q*_max_ is the maximum amount of solute adsorbed per unit weight of adsorbent (μg g^−1^) and *K*_L_ is the Langmuir constant related to the affinity of the binding sites (L μg^−1^).

The Freundlich isotherm model in its linear form was expressed as

(6) $$\text{log}q_{e}=\text{log}K_{F}+\frac{1}{n}\text{log}c_{e}$$

where *K*_f_ is a Freundlich constant indicative of adsorption capacity $\left(\frac{\mu g}{g}{\left(\frac{L}{\mu }\right)}^{\frac{1}{n}}\right)$ and *n* is a dimensionless constant indicating the intensity of the adsorption process.

These models were used to evaluate the adsorption capacity and efficiency of the sponges across various PFOS concentrations and immersion times.

##### Passing‐Through PFOS Solution through BSA‐Based Sponges in a Column

A 3 mL solution containing PFOS, diluted in TRIS to final concentrations of 1000 ppb, was passed through a column packed with one or three BSA‐based sponges. These sponges were prepared using varying concentrations of TW‐20 (5 and 20 mM, 15 K rpm, 2.5 min). The passing‐through of the PFOS solution was conducted over several cycles (1, 3, and 6) to measure the sponges’ PFOS adsorption capacity while maintaining the volume of the solution (3 mL) after each filtration cycle. Subsequently, the filtrate solution was examined by LC‐MS/MS, and the removal efficiency was determined as previously described. Each experiment was repeated three times for each TW‐20 concentration.

##### XPS Analysis

The chemical bonding of BSA‐based sponges before and after PFOS adsorption was analyzed using XPS. For this analysis, sponges were prepared with 20 mM TW‐20 and mixed at 15 K rpm for 2.5 min. One set of sponges was washed with ddH_2_O and freeze‐dried for 2 days. Another set was soaked in a 1000 ppb PFOS solution for 24 h at room temperature with stirring, followed by 24 h without stirring. These sponges were then washed with ddH_2_O for 2 h to remove any unadsorbed PFOS and subsequently freeze‐dried for 2 days. The dried sponges were ground into a fine powder using a pestle, obtaining BSA and BSA‐PFOS powders. Measurements were conducted in an ultrahigh vacuum analysis chamber maintained at 2 × 10^−10^ Torr, using a Versaprobe III‐PHI instrument (PHI, USA). Samples were irradiated with focused monochromated Al Kα X‐rays (1486.6 eV) using a beam size of 100 microns at 25 W and 15 kV. Emitted photoelectrons were directed to a spherical capacitor analyzer. Sample charging was compensated using a dual‐beam charge neutralization system combining a traditional electron flood gun with a low‐energy argon ion beam. Survey spectra were recorded with a pass energy (PE) of 224 eV, a step size of 0.8 eV, and a dwell time of 20 ms, from which the surface chemical composition was determined. The core‐level‐binding energies of different peaks were normalized by setting the binding energy of the C1s peak at 284.8 eV. High‐resolution XPS lines for the elements C1s, O1s, N1s, S2p, and F1s were collected at a PE of 55 eV, a step size of 0.1 eV, and a dwell time of 20 ms. It is important to note that the detection limit of the XPS technique varies between 0.1% and 1% atomic percent. The accuracy of the atomic percent determination depends on the background level, spectral overlap, and overlap with Auger peaks.

## Conflict of Interest

The authors declare no conflict of interest.

## Author Contributions

**Maria Kaeek**: methodology (lead); writing—original draft (equal); writing—review & editing (equal). **Yair Rajmiel**: formal analysis (supporting); writing—review & editing (supporting). **Ofek Goldreich**: formal analysis (supporting); methodology (supporting). **Luai R. Khoury**: conceptualization (lead); supervision (lead); writing—original draft (lead); writing—review & editing (lead).

## Supporting information

Supplementary Material

## Data Availability

The data that support the findings of this study are available from the corresponding author upon reasonable request.
