# Supplementary material for: Bovine Serum Albumin‐Based Sponges as Biocompatible Adsorbents: Development, Characterization, and Perfluorooctane Sulfonate Removal Efficiency
Source: Small Sci. 2025 Mar 2;5(4):2400497. doi: 10.1002/smsc.202400497 (PMC12245007; doi:10.1002/smsc.202400497)
Supplement: Supplementary file 1 — Supplementary Material [file SMSC-5-2400497-s001.zip › smsc12711-sup-0002-SuppData-S2.pdf]

Supporting Information

BSA-Based Sponges as Biocompatible Adsorbents: Development, Characterization, and PFOS Removal Efficiency

Maria Kaeek[1], *Yair Rajmiel*[1], *Ofek Goldreich*[1] *Luai R. Khoury* [1*]

[1] Department of Materials Science and Engineering, Technion Israel Institute of Technology, Haifa, 32000, Israel

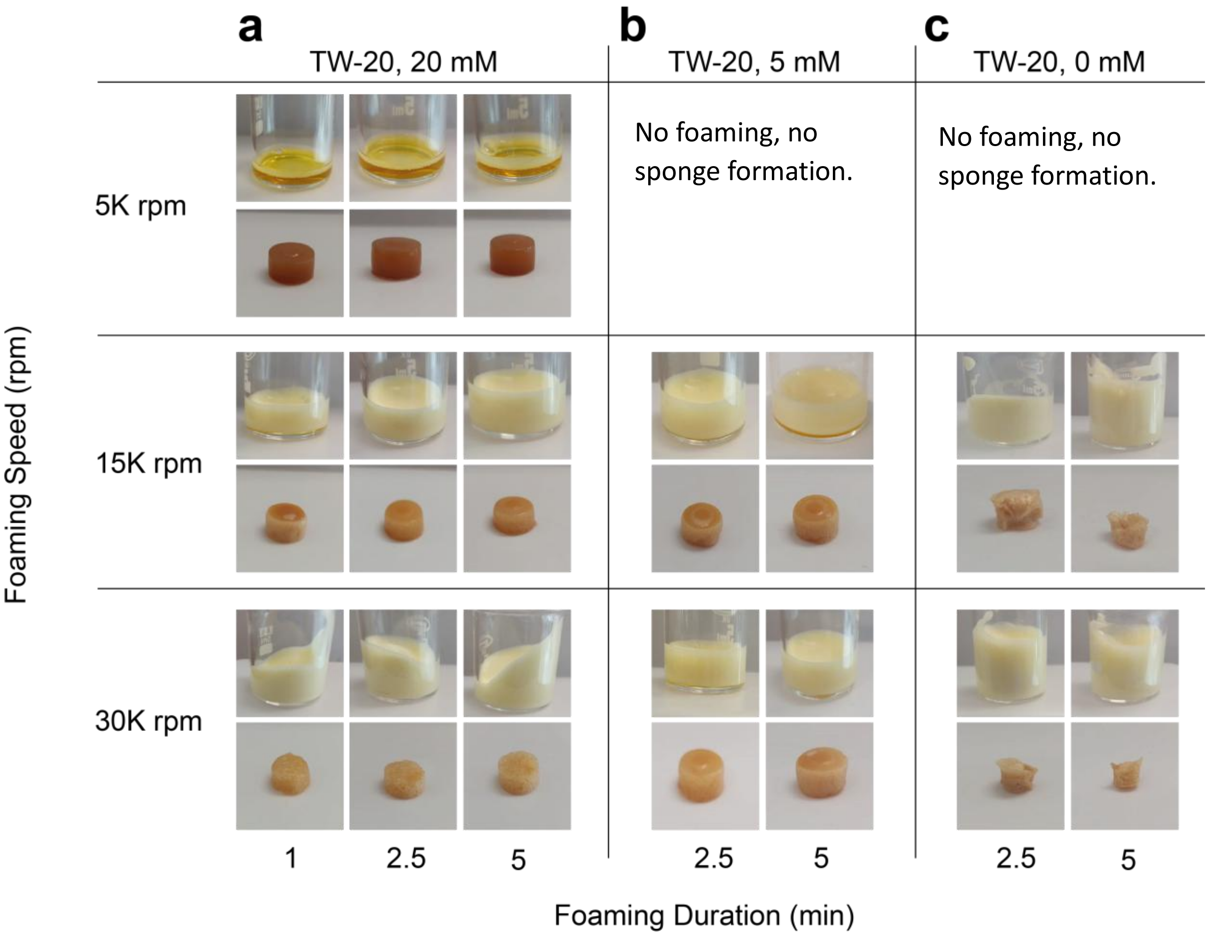

Figure S1. Close-up comparisons of BSA-based sponge morphologies before and after gelation with varying concentrations of TW-20 under different foaming conditions. The top row of images shows the sponges post-foaming, and the bottom row shows the sponges post-crosslinking. Sponges were prepared using a mixture of TW-20, BSA, APS, and Ru(II)bpy$_3^{2+}$, with TW-20 concentrations set to (a) 20 mM, (b) 5 mM, and (c) 0 mM. The mixtures were homogenized at room temperature at

speeds of 5K, 15K, and 30K rpm for durations of 1, 2.5, and 5 minutes. **(a)** TW-20 20 mM: Increasing mixing speeds from 5K to 30K rpm enhanced foam volume. At 5K rpm, inadequate foaming resulted in poor sponge structure across all durations. At 15K rpm, the 1-minute mix led to incomplete foam stabilization, forming a gel layer at the base. Extending mixing to 2.5 and 5 minutes improved the sponge structure. At 30K rpm, the foam became unstable, causing structural weaknesses in the sponges regardless of mixing time. **(b)** TW-20 5 mM: Due to poor results, 5K rpm and 1-minute mixing were omitted. At 30K rpm, excess liquid drained, resulting in sponges with lower liquid content and minimized gel formation at the base. **(c)** TW-20 0 mM: Foam formation was more efficient, with small, densely packed bubbles outperforming the 5 mM concentration due to BSA's surface activity. However, sponges exhibited reduced structural integrity, suffering degradation across all speeds and durations, limiting their practical applicability.

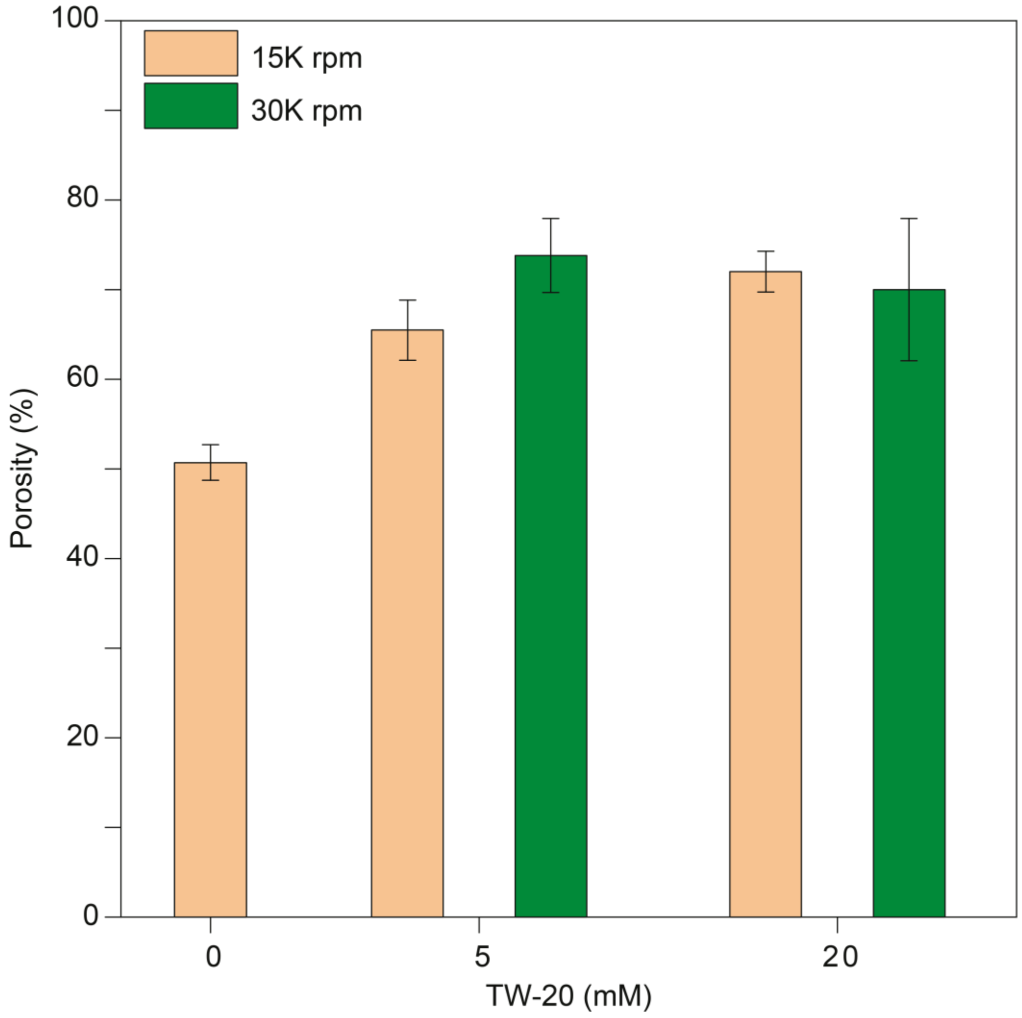

Figure S2. Porosity Variation in BSA-Based Sponges as a Function of TW-20 Concentration and Foaming Speed (Micro-CT Analysis). The porosity of sponges was evaluated using micro-computed tomography (micro-CT), revealing the effects of different conditions. Sponges prepared without TW-20 and mixed at 15K rpm for 2.5 minutes exhibited the lowest porosity, around 51±2%, indicating a tightly packed hydrogel structure. The introduction of 5 mM TW-20 resulted in a slight increase in porosity. A significant rise, up to approximately 72±2%, was observed with 20 mM TW-20, indicating a more open, porous structure. Increasing the mixing speed to 30K rpm with 5 mM TW-20 further enhanced porosity. However, when both mixing speed was increased to 30K rpm and TW-20 concentration was raised to 20 mM, porosity decreased to around 70±8%. This suggests that higher mixing speeds, in combination with elevated surfactant concentrations, may compromise foam stability, potentially leading to pore collapse and a less porous protein network.

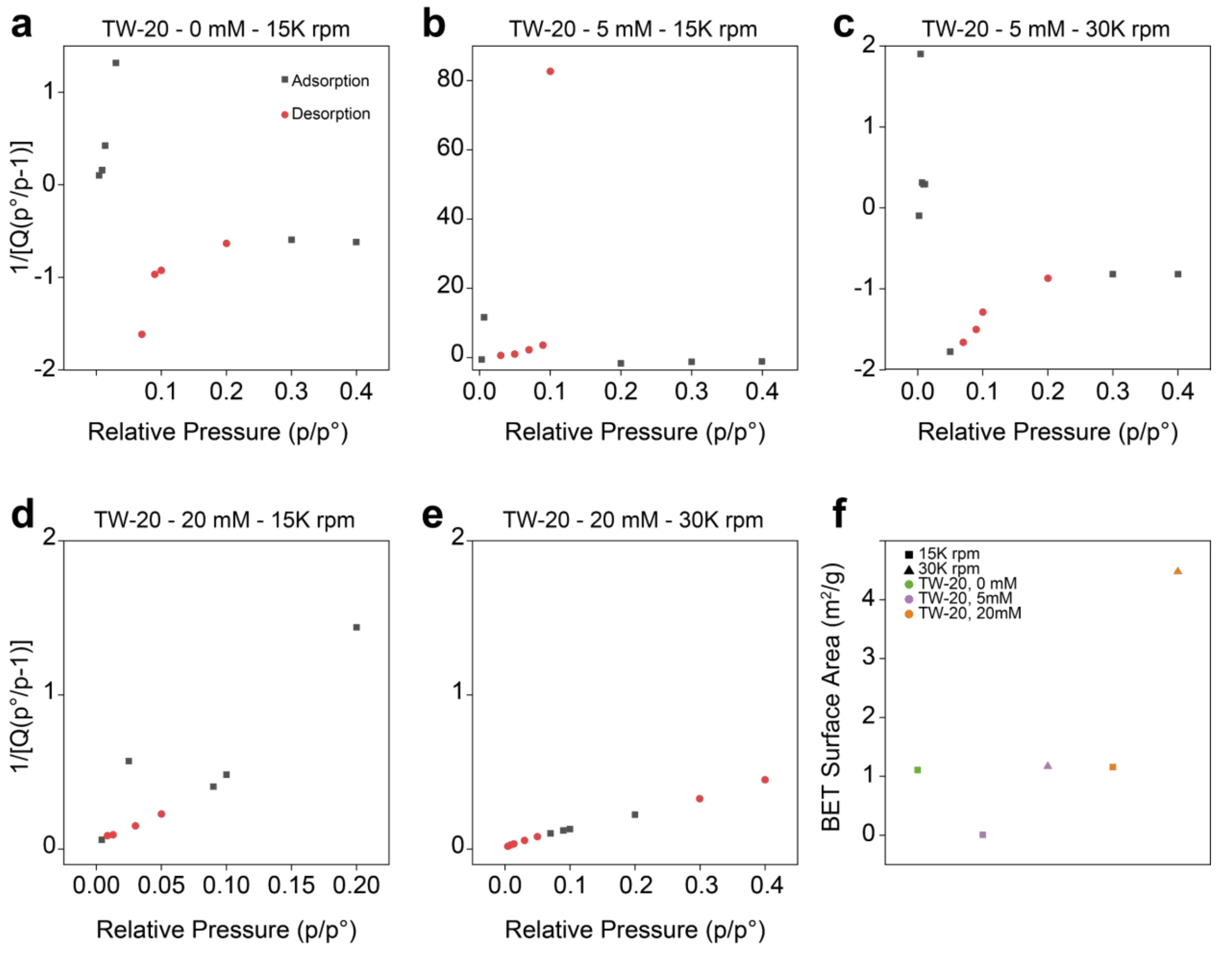

Figure S3. Brunauer–Emmett–Teller (BET) Surface Area Plots of BSA-Based Sponges Prepared with Various TW-20 Concentrations and Foaming Speeds (Foaming Duration: 2.5 Minutes). The BET model was applied to determine the surface area of the sponges based on adsorption and desorption isotherms. The plots (a-e) illustrate the relationship between relative pressure (P/P_0) and $1/[Q(P_0/P) - 1]$, where Q represents the quantity of gas adsorbed at a given relative pressure. (a) TW-20, 0 mM-15K rpm: Scattered adsorption (black) and desorption (red) points indicate poor surface area measurements and an inconsistent pore structure. (b) TW-20, 5 mM-15K rpm: While there is a slight trend in the adsorption data, the overall scattering suggests suboptimal surface area measurements. (c) TW-20, 5 mM-30K rpm: Similar to 15K rpm, no clear linear trend is observed, indicating limited surface area and inconsistent pore formation. (d) TW-20, 20 mM-15K rpm: A well-defined linear trend emerges, suggesting a measurable surface area and a more consistent pore structure. (e) TW-20, 20 mM-30K rpm: A linear trend is evident, indicating measurable surface area. (f) BET Surface Area Calculations: The surface area increases with higher TW-20 concentrations,

peaking at 20 mM TW-20 with 30K rpm. This trend reflects the role of both TW-20 concentration and foaming speed in enhancing surface area through increased pore formation.

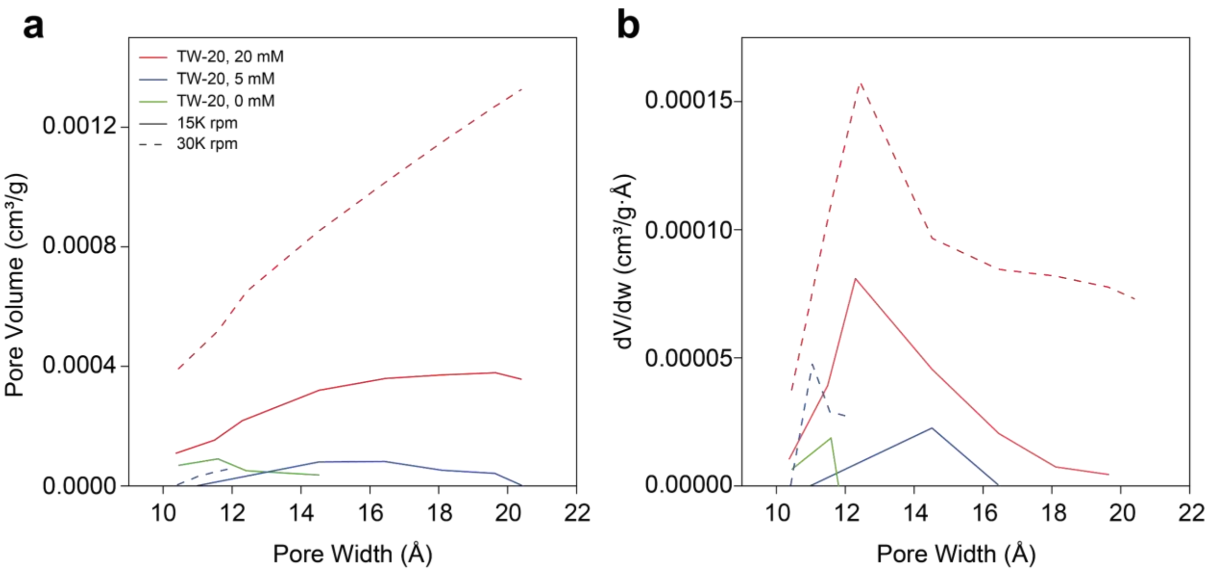

Figure S4. Horvath-Kawazoe (HK) Micropore Analysis of BSA-Based Sponges Prepared with Various TW-20 Concentrations and Foaming Speeds (Foaming Duration: 2.5 Minutes). (a) Pore Volume vs. Pore Width: The solid red line represents sponges prepared with 20 mM TW-20 at 15K rpm, while the dashed red line represents 20 mM TW-20 at 30K rpm. These sponges displayed significant pore volumes, with the 30K rpm samples showing higher values across the entire pore width range. Both foaming speeds for the 20 mM TW-20 samples exhibited a peak pore volume around 12 Å, indicating a substantial presence of micropores. The solid blue line represents 5 mM TW-20 at 15K rpm, and the dashed blue line represents 5 mM TW-20 at 30K rpm, both of which showed much lower pore volumes, with a slight increase at 15K rpm. The solid green line represents sponges prepared without TW-20 at 15K rpm, showing the lowest pore volumes, indicating very limited micropore formation. (b) Differential Pore Volume (dV/dw) vs. Pore Width: The solid red line (20 mM TW-20, 15K rpm) and dashed red line (20 mM TW-20, 30K rpm) both exhibited prominent peaks at around 12 Å, with the 30K rpm sample showing a higher peak, suggesting a greater density of micropores. For the 5 mM TW-20 samples, the solid blue line (15K rpm) showed a slightly higher peak than the dashed blue line (30K rpm), indicating a lower density of micropores. The solid green line (0 mM TW-20) exhibited minimal peaks, confirming poor micropore formation.

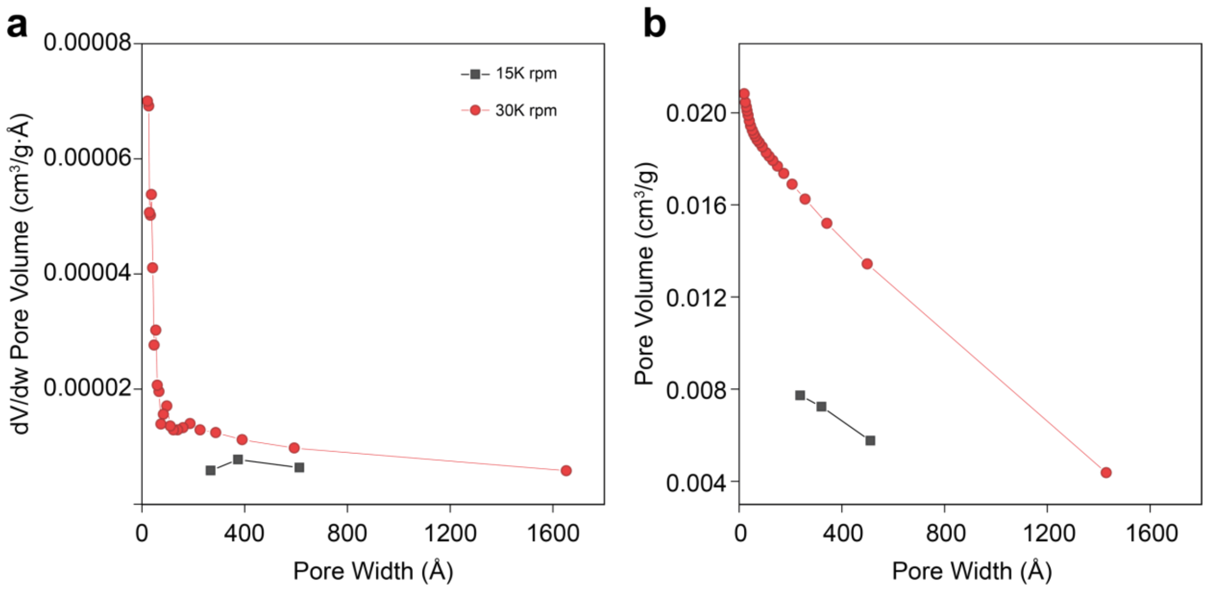

Figure S5. Barrett-Joyner-Halenda (BJH) Mesopore Analysis of BSA-Based Sponges Prepared with 20 mM TW-20 at 15K and 30K rpm Foaming Speeds (Foaming Duration: 2.5 Minutes). (a) Differential Pore Volume (dV/dW) vs. Pore Width: The sample foamed at 30K rpm exhibits a significantly higher differential pore volume compared to the 15K rpm sample, particularly at smaller pore widths (0–200 Å). The 30K rpm sample also maintains elevated pore volumes up to a pore width of approximately 1600 Å, indicating a broader distribution of mesopores. (b) Pore Volume vs. Pore Width: The 30K rpm sample demonstrates a higher total pore volume across the entire pore width range compared to the 15K rpm sample. The increased pore volume at smaller pore widths (0–200 Å) in the 30K rpm sample suggests a greater density of mesopores, contributing to a more extensive and well-developed mesoporous network.

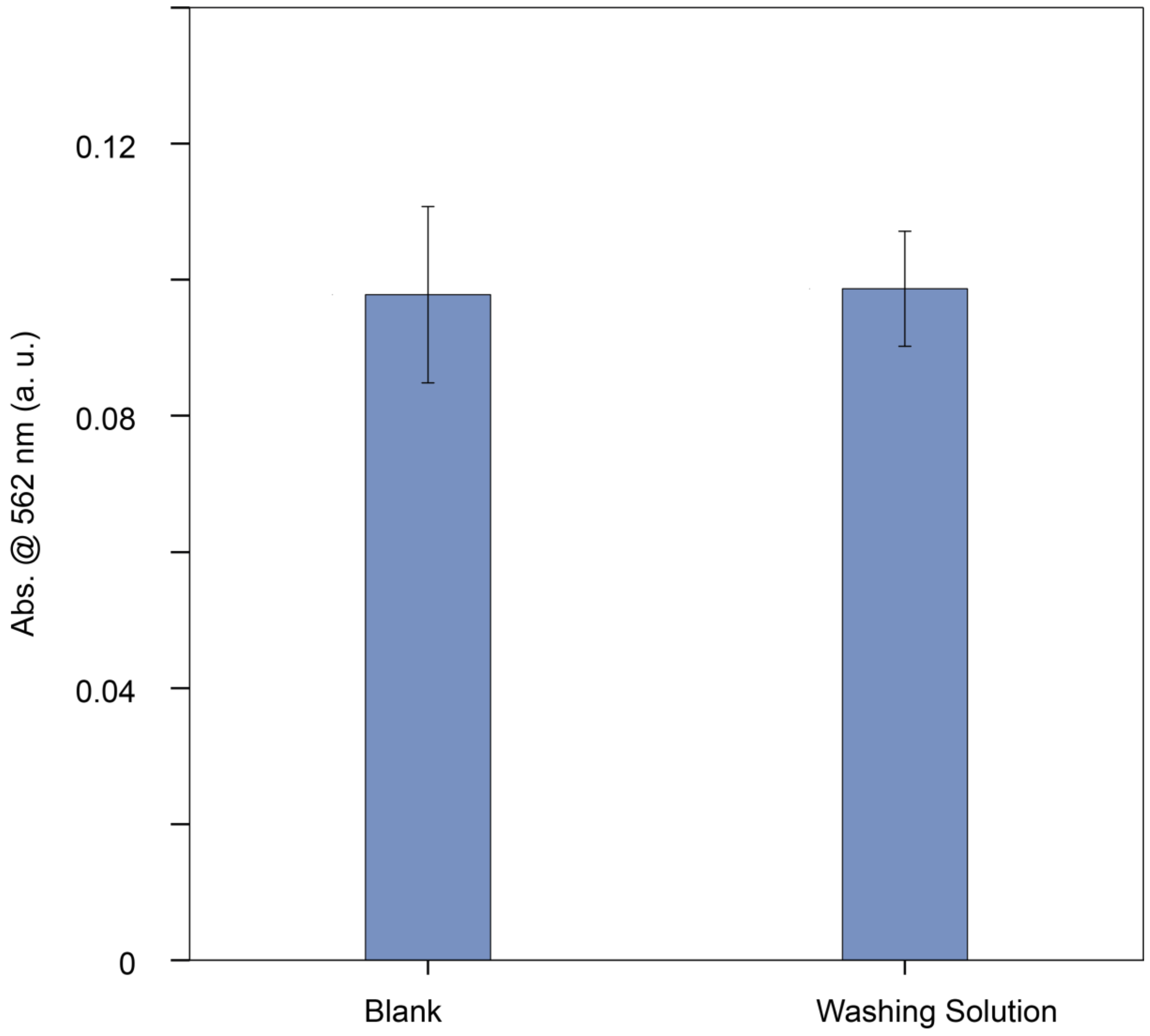

Figure S6. Evaluating Protein Release from BSA-Based Sponges Using the Bicinchoninic Acid (BCA) Assay. This assay was employed to assess the structural stability of BSA-based sponges during extended exposure to TRIS buffer. Over a 6-hour period, the sponges were washed with TRIS, refreshing the solution every two hours. From these washing solutions, 25 μL samples were extracted for protein content analysis using the BCA Protein Assay. The absorbance measured at 562 nm showed negligible differences between the washing solution samples and the Blank (TRIS). This minimal variance in absorbance suggests that the sponges exhibit robust structural integrity with almost no protein leaching.

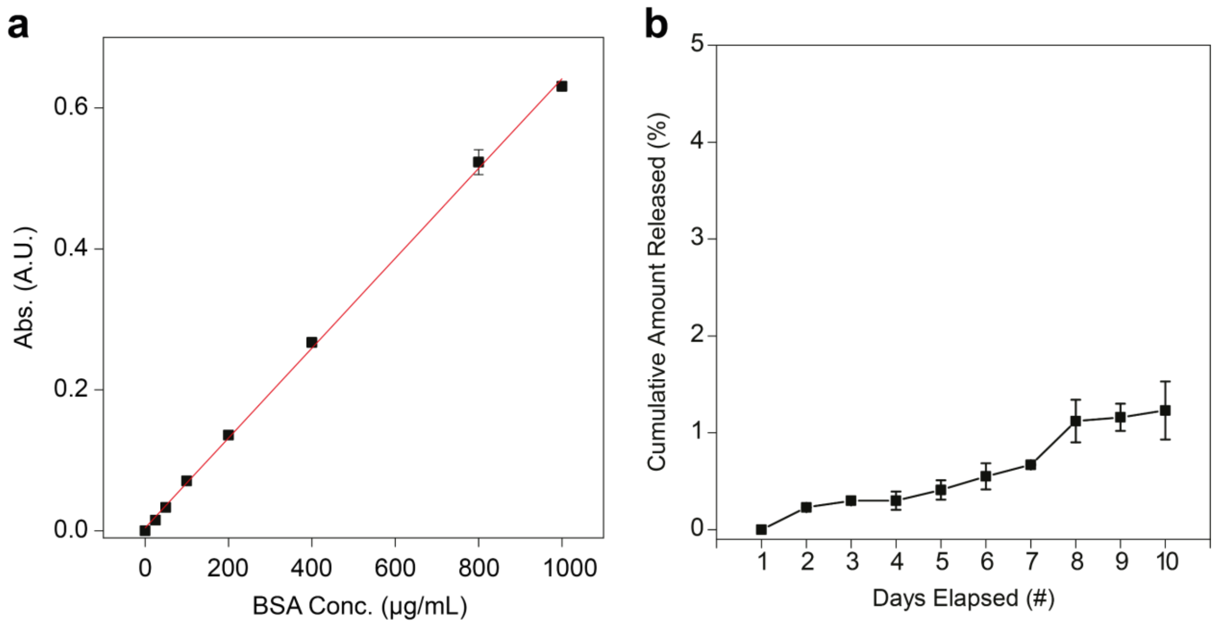

Figure S7. BSA leaching analysis and sponge stability. (a) Calibration curve for BSA concentration using the BCA assay, used to quantify the total amount of BSA released from the sponge over time. (b) Cumulative amount of BSA from BSA-based sponges soaked in TRIS buffer (pH ~7.4) over 10 days. The release profile shows a small cumulative release pattern, with gradual increases over time, reaching 1.16 ± 0.14% by day 10. These results confirm the structural stability of the sponges under prolonged aqueous conditions.

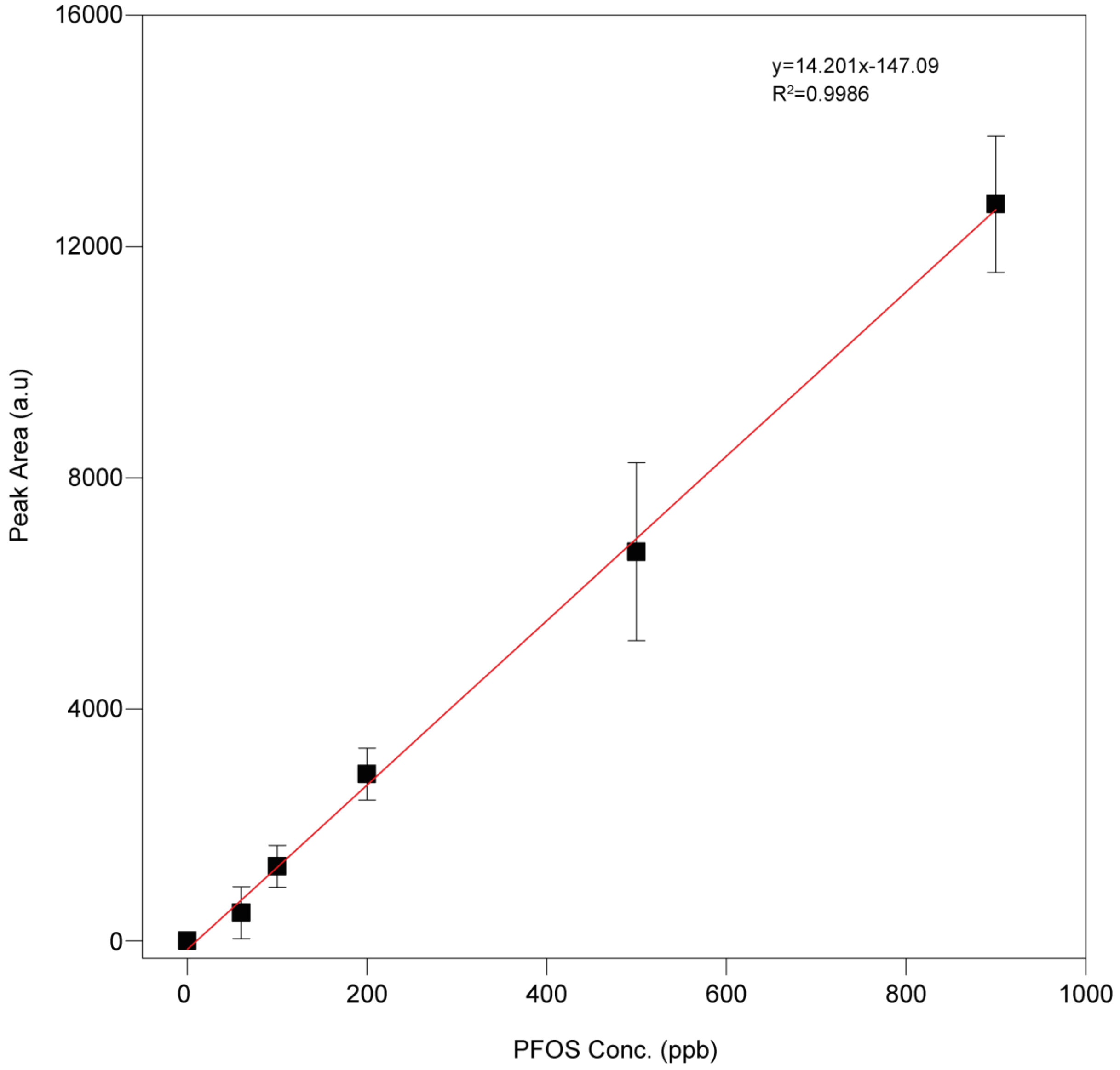

Figure S8. Calibration Curve for PFOS Quantification Using High-Performance LC-MS/MS. This figure displays the calibration curve, which plots the peak area (arbitrary units, A.U.) against a range of PFOS concentrations (ppb). The peak area corresponds to the signal intensity detected for PFOS, representing the response of ionized PFOS molecules during the mass spectrometry (MS) process.

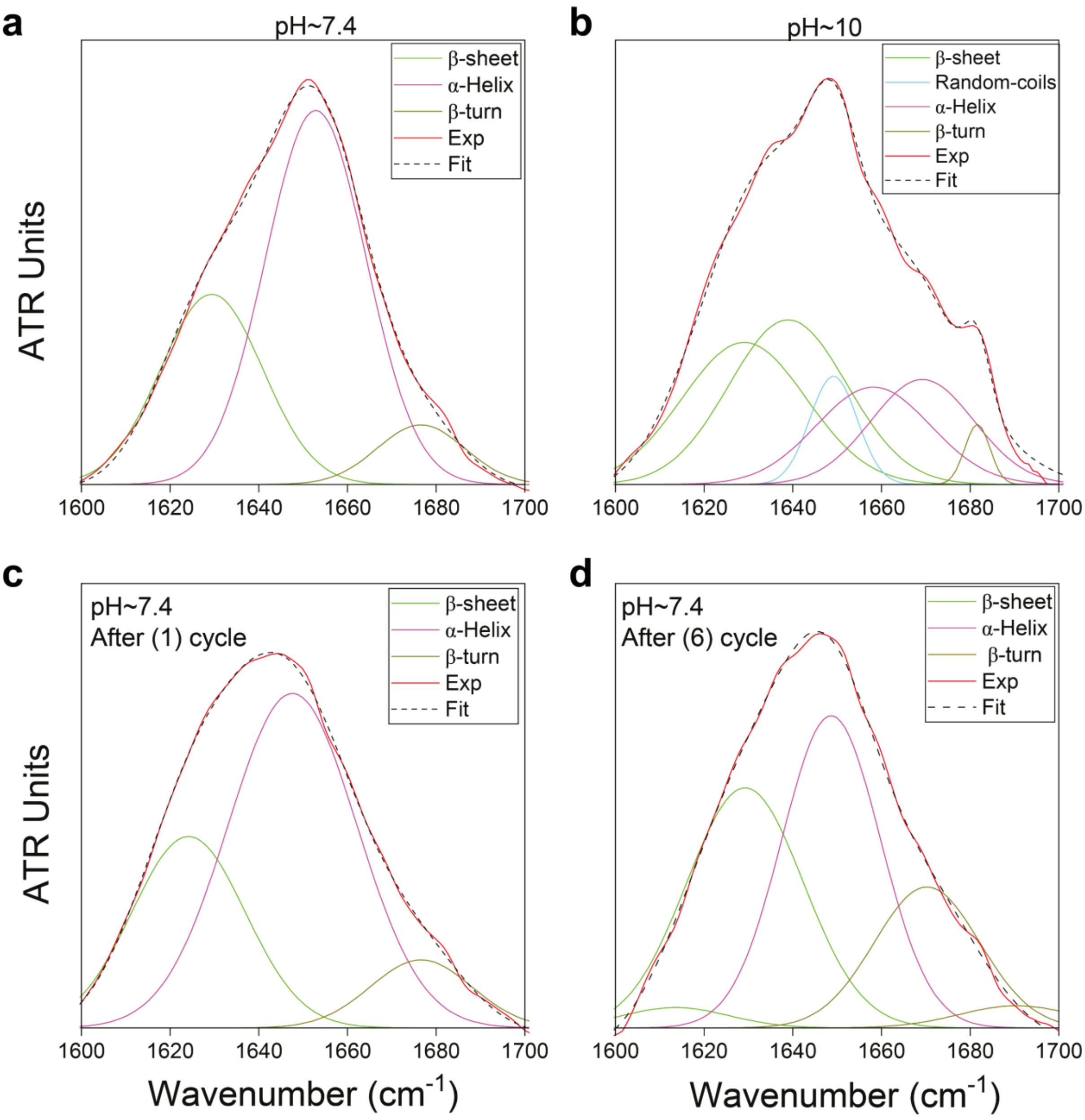

Figure S9. Changes in the secondary structure of BSA-based sponges during adsorption-desorption cycles. (a) Secondary structure of BSA-based sponge, where α-helix content makes up the largest portion of BSA, with 63.4 ± 2.9%, β-sheet content of 29.05 ± 1.9% and β-turn content of 7.5 ± 1.0%. (b) Washing at pH~10 induced significant changes in the secondary structure, increasing the β-sheet content to 49.6 ± 8.6%, while α-helix content decreased to 40.3 ± 0.7% and β-turn content decreased to 4.3 ± 0.3%. These changes reflect structural rearrangements due to disruption of non-covalent interactions, such as hydrogen bonding and electrostatic forces, in the alkaline environment. (c) Washing the sponges with TRIS buffer at pH ~7.4 after the first desorption cycle neutralized the

residual alkaline solution and restored the BSA structure, with α-helix content increasing to 59.9 ± 4.9% ,β-sheet content decreasing to 25.9 ± 3.0%, and β-turn content increasing to 9.8 ± 0.3%. (d) After the sixth desorption cycle, washing with TRIS buffer at pH ~7.4 did not fully recover the α-helix structure, with α-helix content restored only to 44.2 ± 5.4%, β-sheet content decreasing only to 41.8 ± 2.9%, and β-turn content increasing to 19.7 ± 2.8%. This incomplete recovery of the α-helix and β-structures after repeated cycles may be attributed to the progressive exposure to alkaline pH, which disrupt the protein's native conformation and prevent the complete restoration of its original secondary structure. These results emphasize the dynamic nature of the sponge's secondary structure in response to pH changes, which is crucial for maintaining its functionality during reusability experiments.

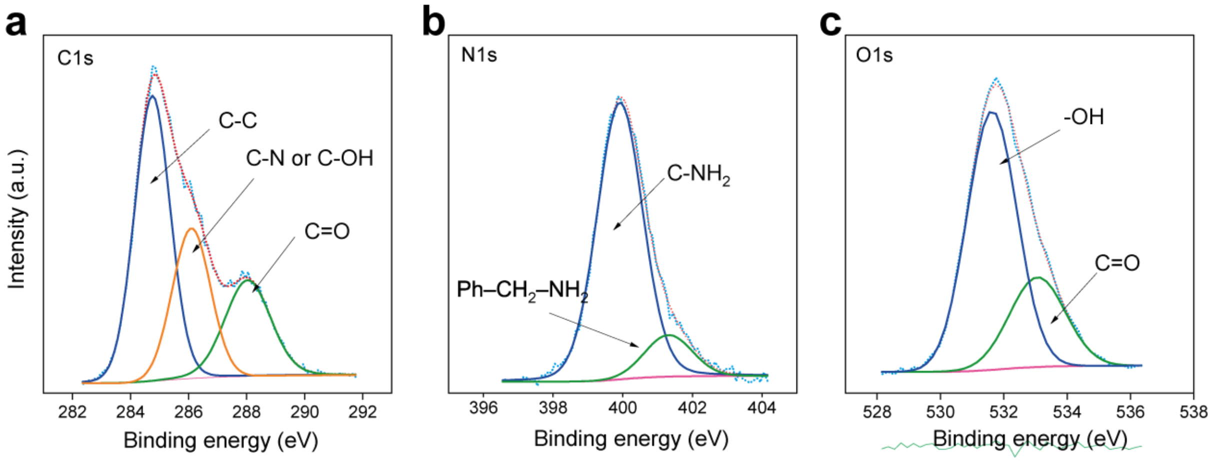

Figure S10. XPS Spectra of BSA-based sponge Without PFOS. (a) C1s Spectrum: Three distinct peaks represent key chemical functionalities in BSA. The 284.8 eV peak (blue) corresponds to C–C bonds, characteristic of aliphatic and aromatic carbon-carbon structures. The 286.1 eV peak(orange) is attributed to C–N and C–OH bonds, indicative of amines or hydroxyl groups within the BSA structure. The 288.0 eV peak (green) signifies C=O bonds, associated with carbonyl groups in carboxyl and amide functionalities. **(b)** N1s Spectrum: The 400.0 eV peak (blue) corresponds to C–NH_2 bonds, representing amine groups in BSA. The 401.3 eV peak (green) is attributed to nitrogen in the phenylalanine side chain, an aromatic amino acid. **(c)** O1s Spectrum: The 531.6 eV peak (blue) corresponds to hydroxyl (–OH) groups, found in serine and threonine residues. The 533.1 eV peak (green) is associated with carboxyl (–COOH) groups, present in aspartic and glutamic acid

residues. These XPS findings confirm the presence of hydroxyl, amine, carboxyl, and carbonyl groups in BSA, providing critical insights into its surface chemistry and functional interactions.

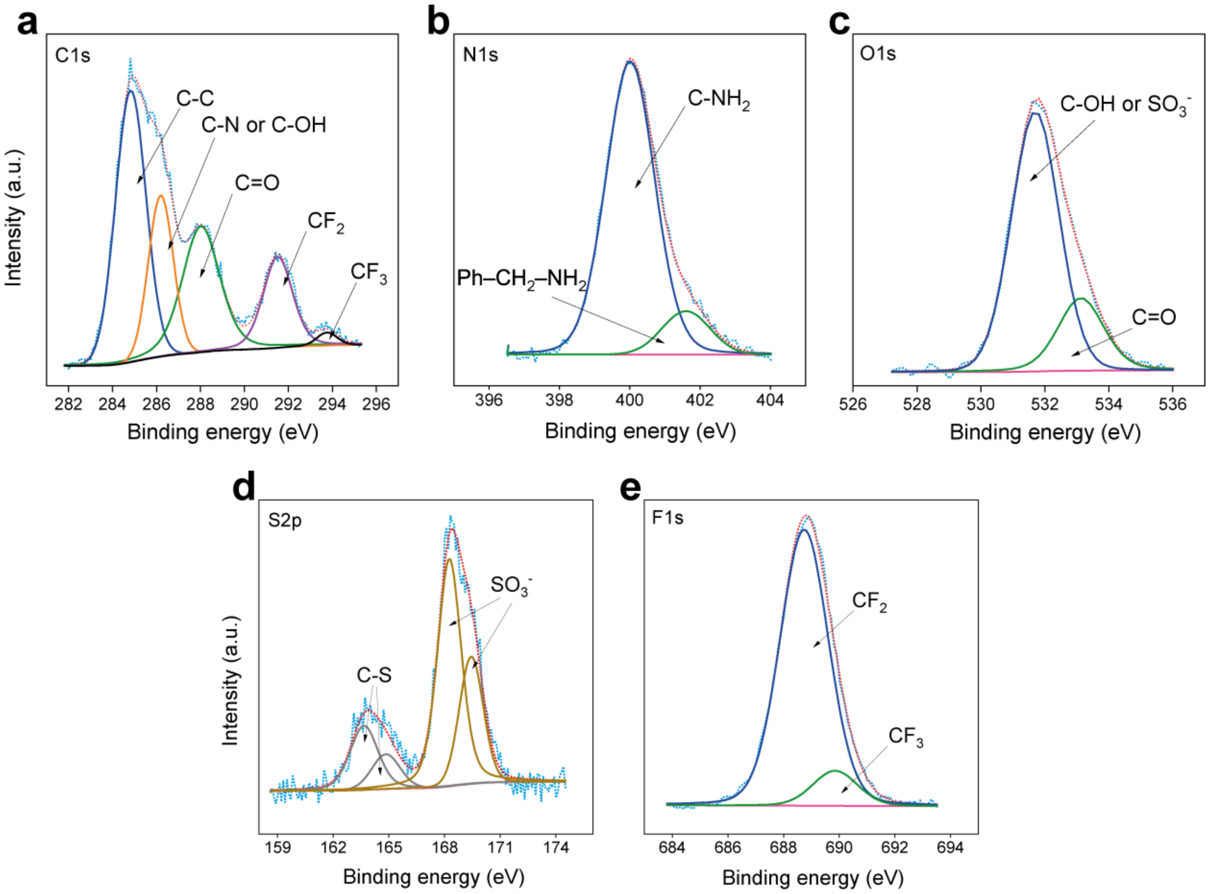

Figure S11. XPS Spectra of BSA-Based Sponges After PFOS Adsorption. XPS analysis reveals changes in chemical bonding environments in the C1s, N1s, O1s, S2p, and F1s regions following PFOS adsorption. (a) C1s Spectrum: Five distinct peaks correspond to key functional groups. The 284.8 eV peak (blue) represents C–C bonds, commonly found in the BSA backbone and aromatic rings. The 286.2 eV peak (orange) corresponds to C–S, C–N, or C–OH bonds, indicating sulfonate, amine, or hydroxyl groups. The 288.0 eV peak (green) is associated with C=O functional groups, likely from carboxyl moieties in BSA. The 291.5 eV peak (purple) corresponds to CF_2 groups, confirming fluorinated carbon from PFOS. The 293.8 eV peak (black) represents CF_3 groups, further verifying PFOS adsorption onto the BSA-based sponge. **(b)** N1s Spectrum: The 400.0 eV peak (blue) corresponds to C–NH_2 bonds, indicative of amine groups in BSA, particularly from lysine residues. The 401.6 eV peak (green) corresponds to phenylalanine, confirming the presence of this aromatic amino acid in the BSA structure. **(c)** O1s Spectrum: The 531.7 eV peak (blue) corresponds

to hydroxyl (C–OH) groups from BSA or sulfonate ($–SO_3^-$) groups from PFOS. The 533.1 eV peak (green) is associated with C=O groups, confirming the presence of carboxyl functional groups in BSA. **(d)** S2p Spectrum: Two doublets are observed, each separated by a spin-orbit splitting of 1.18 eV. The 163.7 eV peak(gray) corresponds to C–S bonds, indicative of sulfur-containing linkages in BSA. The 168.3 eV peak (brown) is attributed to sulfonate ($–SO_3^-$) groups, confirming PFOS adsorption on the BSA-based sponge. **(e)** F1s Spectrum: The 688.7 eV peak (blue) and 689.9 eV peak (green) correspond to CF_2 and CF_3 groups, respectively, confirming the presence of fluorinated PFOS components on the BSA-based sponge. These XPS findings provide clear evidence of PFOS adsorption onto the BSA-based sponge through hydrophobic, electrostatic, and sulfur interactions, highlighting key functional group modifications.

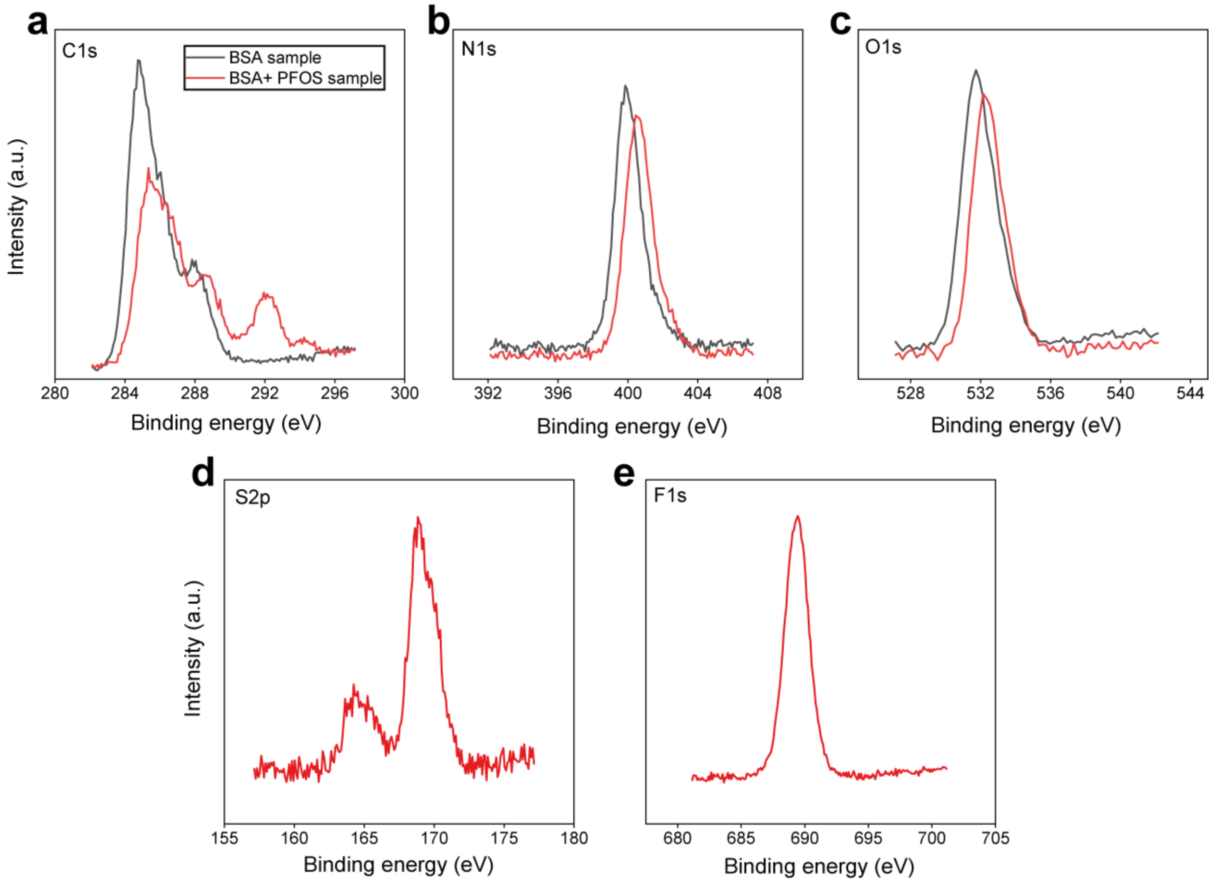

Figure S12. XPS Analysis: Superposition of C1s, N1s, O1s, S2p, and F1s Spectra for BSA and BSA+PFOS-Based Sponges. **(a)** C1s Spectrum: The C1s spectra for BSA (black line) and BSA+PFOS (red line) reveal a +0.7 eV shift toward higher binding energies for C–C bonds in the BSA+PFOS sample. This shift suggests hydrophobic interactions between the fluorinated

tail of PFOS and aromatic residues in BSA, such as phenylalanine. Additionally, peaks at ~292 eV and ~295 eV, corresponding to CF_2 and CF_3 groups in PFOS, confirm the presence of adsorbed fluorinated moieties, with binding energy shifts reflecting hydrophobic interactions. **(b)** N1s Spectrum: The N1s spectra exhibit a shift in the C–NH_2 peak at 400.0 eV upon PFOS adsorption, indicating interactions between amine groups (from lysine and arginine) and PFOS. The +0.7 eV shift suggests electrostatic interactions between the negatively charged sulfonate group of PFOS and the positively charged amine groups of BSA. **(c)** O1s Spectrum: A +0.6 eV shift is observed at 531.7 eV, corresponding to hydroxyl groups in BSA or sulfonate groups in PFOS. This shift suggests that PFOS adsorption alters the oxygen environment, with hydroxyl groups from serine or threonine engaging in hydrogen bonding with the sulfonate group of PFOS, leading to electron density modifications around oxygen atoms. **(d)** S2p Spectrum: The BSA+PFOS spectrum exhibits two doublets. The first peak at 163.7 eV corresponds to C–S bonds in BSA, likely from cysteine residues. The second peak at 168.3 eV is characteristic of the sulfonate group in PFOS, confirming its adsorption onto the BSA-based sponge and direct involvement in sulfur-based interactions. **(e)** F1s Spectrum: A prominent peak at 689 eV corresponds to CF_2 and CF_3 groups, verifying the presence of fluorinated PFOS moieties on the BSA-based sponge. These findings confirm hydrophobic interactions between the fluorinated tail of PFOS and the non-polar aromatic rings of BSA.

Video S1. 3D micro-CT visualization of BSA-based sponges. This video demonstrates the macrostructure of BSA-based sponges, prepared with 20 mM TW-20, and analyzed after Lugol staining to enhance contrast. The non-destructive micro-CT scan reveals details of porosity, density, voids, and fiber orientation, providing an in-depth look at the sponge morphology. The video also captures different cross-sectional views as we penetrate into the sponge, revealing the intricate pore distribution and variations in the internal structure.

Video S2. Absorption and Filtration Capabilities of BSA-Based Sponges for Water Purification. This video demonstrates the ability of BSA-based sponges to absorb and filter contaminants from water. A BSA-based sponge is soaked in a water solution with blue dye to simulate polluted water. As the sponge absorbs the colored water and is squeezed, the dye molecules remain on the sponge while

clear water is released, highlighting the sponge’s adsorption properties. Additionally, the video highlights the remarkable shape memory of the BSA-based sponge, as it returns to its original form when placed back in the water after being squeezed, further illustrating its structural resilience.

.
